# ZapG (YhcB/DUF1043), a novel cell division protein in gamma-proteobacteria linking the Z-ring to septal peptidoglycan synthesis

**DOI:** 10.1016/j.jbc.2021.100700

**Published:** 2021-04-23

**Authors:** Jitender Mehla, George Liechti, Randy M. Morgenstein, J. Harry Caufield, Ali Hosseinnia, Alla Gagarinova, Sadhna Phanse, Norman Goodacre, Mary Brockett, Neha Sakhawalkar, Mohan Babu, Rong Xiao, Gaetano T. Montelione, Sergey Vorobiev, Tanneke den Blaauwen, John F. Hunt, Peter Uetz

**Affiliations:** 1 Center for the Study of Biological Complexity, Virginia Commonwealth University, Richmond, Virginia, USA; 2 Department of Microbiology and Immunology, Henry Jackson Foundation, Uniformed Services University of the Health Sciences, Bethesda, Maryland, USA; 3 Department of Microbiology and Molecular Genetics, Oklahoma State University, Stillwater, Oklahoma, USA; 4 Department of Biochemistry, Research and Innovation Centre, University of Regina, Regina, Saskatchewan, Canada; 5 Department of Biochemistry, College of Medicine, University of Saskatchewan, Saskatoon, Saskatchewan, Canada; 6 Nexomics Biosciences Inc., Rocky Hill, New Jersey, USA; 7 Department of Chemistry and Chemical Biology, and Center for Biotechnology and Interdisciplinary Sciences, Rensselaer Polytechnic Institute, Troy, New York, USA; 8 Department of Chemistry and Chemical Biology, Rensselaer Polytechnic Institute, Troy, New York, USA; 9 Department of Biological Sciences, Columbia University, New York, New York, USA; 10 Bacterial Cell Biology & Physiology, Swammerdam Institute for Life Sciences, University of Amsterdam, Amsterdam, Netherlands

**Keywords:** cell division/divisome, DUF1043/envelope biosynthesis/FtsZ/FtsI, protein function and structure/RodZ/X-ray crystallography, B2H, bacterial two hybrid, CM, cytoplasmic membrane, EDA-DA, ethynyl-D-alanyl-D-alanine, EXP, exponential, NADA, nontoxic, fluorescent D-amino acid analog of D-alanine, OG, orthologous group, ON, overnight, PG, peptidoglycan, PPIs, protein-protein interactions, STAT, stationary, TM, transmembrane.

## Abstract

YhcB, a poorly understood protein conserved across gamma-proteobacteria, contains a domain of unknown function (DUF1043) and an N-terminal transmembrane domain. Here, we used an integrated approach including X-ray crystallography, genetics, and molecular biology to investigate the function and structure of YhcB. The *Escherichia coli yhcB* KO strain does not grow at 45 °C and is hypersensitive to cell wall–acting antibiotics, even in the stationary phase. The deletion of *yhcB* leads to filamentation, abnormal FtsZ ring formation, and aberrant septum development. The Z-ring is essential for the positioning of the septa and the initiation of cell division. We found that YhcB interacts with proteins of the divisome (*e.g.*, FtsI, FtsQ) and elongasome (*e.g.*, RodZ, RodA). Seven of these interactions are also conserved in *Yersinia pestis* and/or *Vibrio cholerae*. Furthermore, we mapped the amino acid residues likely involved in the interactions of YhcB with FtsI and RodZ. The 2.8 Å crystal structure of the cytosolic domain of *Haemophilus ducreyi* YhcB shows a unique tetrameric α-helical coiled-coil structure likely to be involved in linking the Z-ring to the septal peptidoglycan-synthesizing complexes. In summary, YhcB is a conserved and conditionally essential protein that plays a role in cell division and consequently affects envelope biogenesis. Based on these findings, we propose to rename YhcB to ZapG (Z-ring-associated protein G). This study will serve as a starting point for future studies on this protein family and on how cells transit from exponential to stationary survival.

The sequencing revolution has flooded databases with millions of uncharacterized protein sequences. Only 0.8% of the ∼180 million protein sequences in UniProtKB/TrEMBL (1) are experimentally annotated or are associated with transcript data (UniProt, February 2, 2020). Around 25.51% of sequence annotations have been inferred by homology, and another 73.69% of sequences have been annotated by prediction algorithms (1). The functions of most proteins in UniProt (or Pfam) are either computationally predicted or unknown. Therefore, functional characterization of unknown proteins remains a rate-limiting step in molecular biology (2, 3, 4). One of these uncharacterized proteins, *Escherichia coli* YhcB, was initially thought to be a subunit of cytochrome bd (oxidase) but was later found to be dispensable for the assembly of cytochrome bd (5). Large-scale genomic and proteomic studies indicated that *yhcB* may be involved in biofilm formation (6), cell envelope integrity (7), cold sensitivity (8), and DNA repair processes (9, 10, 11). Furthermore, a synthetic lethal phenotype was observed in combination with a deletion of the cell shape maintenance gene *rodZ* (12). The latter study suggested a role in cell division which was recently confirmed by Sung *et al*. (13), who also found cell division defects in *yhcB* deletion strains. However, the molecular mechanism of these phenotypes remained unknown. Here, we investigate the structure and function of *E. coli* YhcB and its role in cell division by using various interaction screens and functional assays. Most importantly, we investigated the molecular basis of these phenotypes by determination of the X-ray crystal structure of the cytoplasmic region of the YhcB ortholog from *Haemophilus ducreyi*.

## Results

### YhcB is conserved in proteobacteria

**Figure 1:**
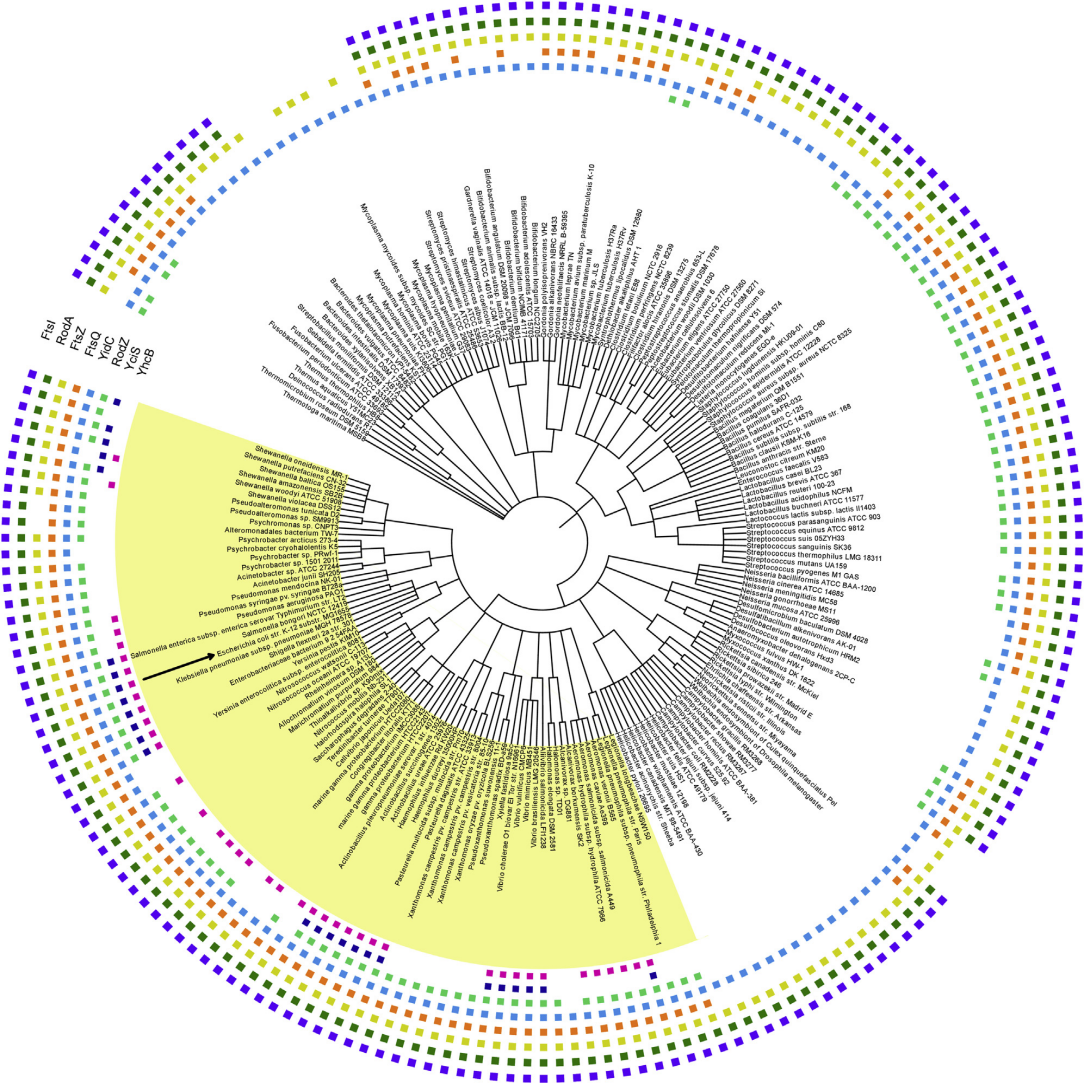
**Phylogenomics ofthe*yhcB* gene.** Phylogenetic profile of YhcB and its interacting proteins. Proteobacteria are highlighted in *yellow*. *Escherichia coli* is indicated by a *black arrow*. Each *square* indicates the presence of the protein in the indicated species/genome. The tree was made using iTOL v4 (56).

YhcB is conserved across most gamma-proteobacteria but absent in other bacterial genomes (Fig. 1). The *yhcB* gene is typically located upstream of two periplasmic outer membrane stress sensor proteases (*degQ* and *degS*) and downstream of a cell division gene *zapE* (*yhcM*), which is encoded on the opposite strand (Fig. S1).

### *yhcB* deletion results in multiple phenotypes

**Figure 2:**
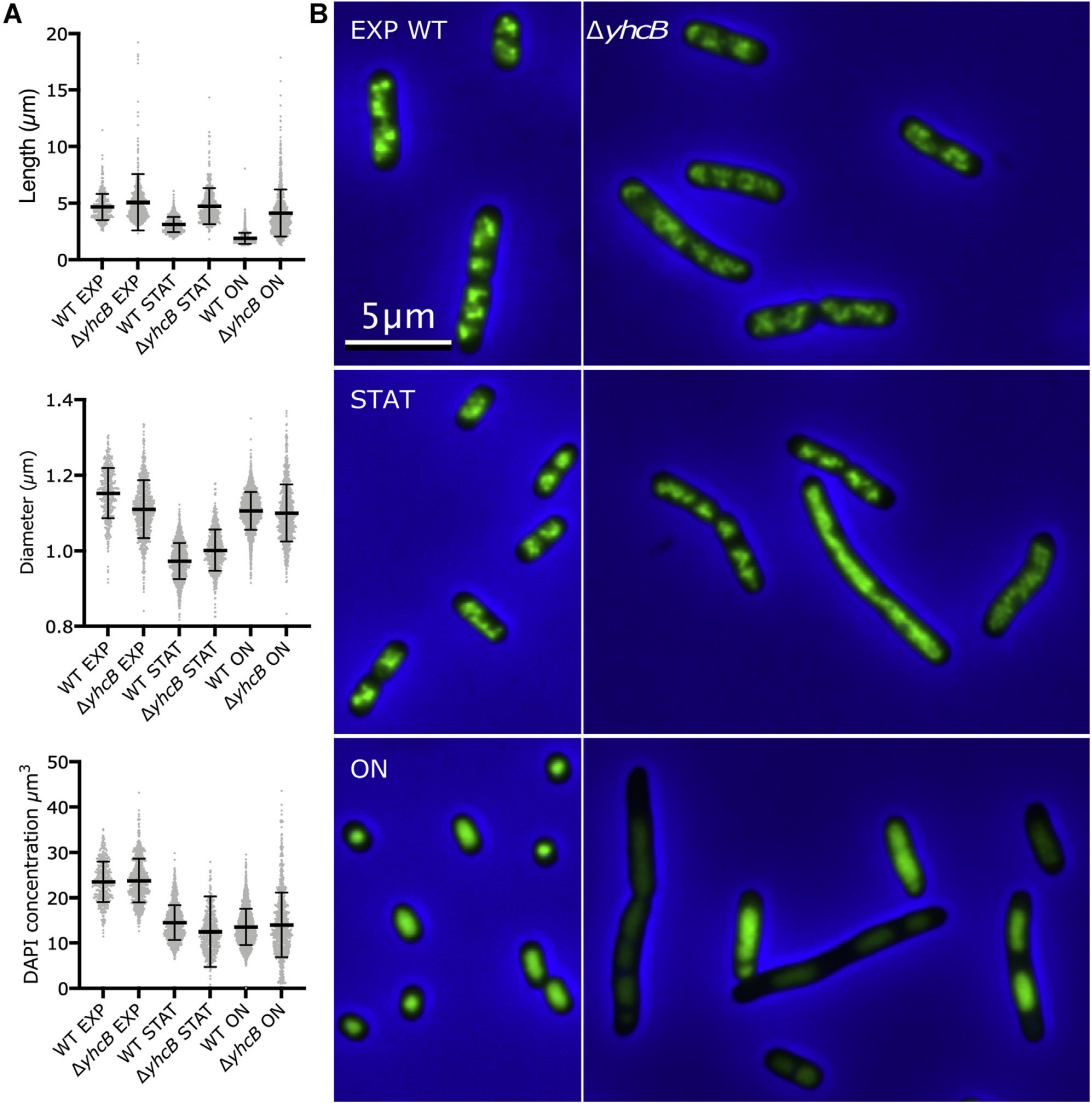
**Δ*yhcB* lacks proper stationary state growth regulation.***A*, the Δ*yhcB* and its parental strain BW25113 (WT) were grown in LB at 37 °C for 24 h (overnight [ON]) and then diluted 1:1000 and grown to an absorbance at 650 nm of 0.3 (EXP[onential]) or to an absorbance at 650 nm of 1.2 (STAT[ionary]) and fixed, and the nucleoids were stained with 4',6-diamidino-2-phenylindole (DAPI). The length, diameter, and DAPI fluorescence of each culture are indicated with the mean and SD. The number of analyzed WT cells was 434 (EXP), 1225 (STAT), and 2133 (ON), respectively. The number of analyzed Δ*yhcB* cells was 555 (EXP), 776 (STAT), and 811 (ON), respectively. *B*, representative images form all six cultures with WT on the left and Δ*yhcB* on the right. The images are merged phase-contrast (*gray*) and DAPI (*green*) images with a *blue* background for optimal contrast. Brightness, contrast, and scale are the same for all images.

**Figure 3:**
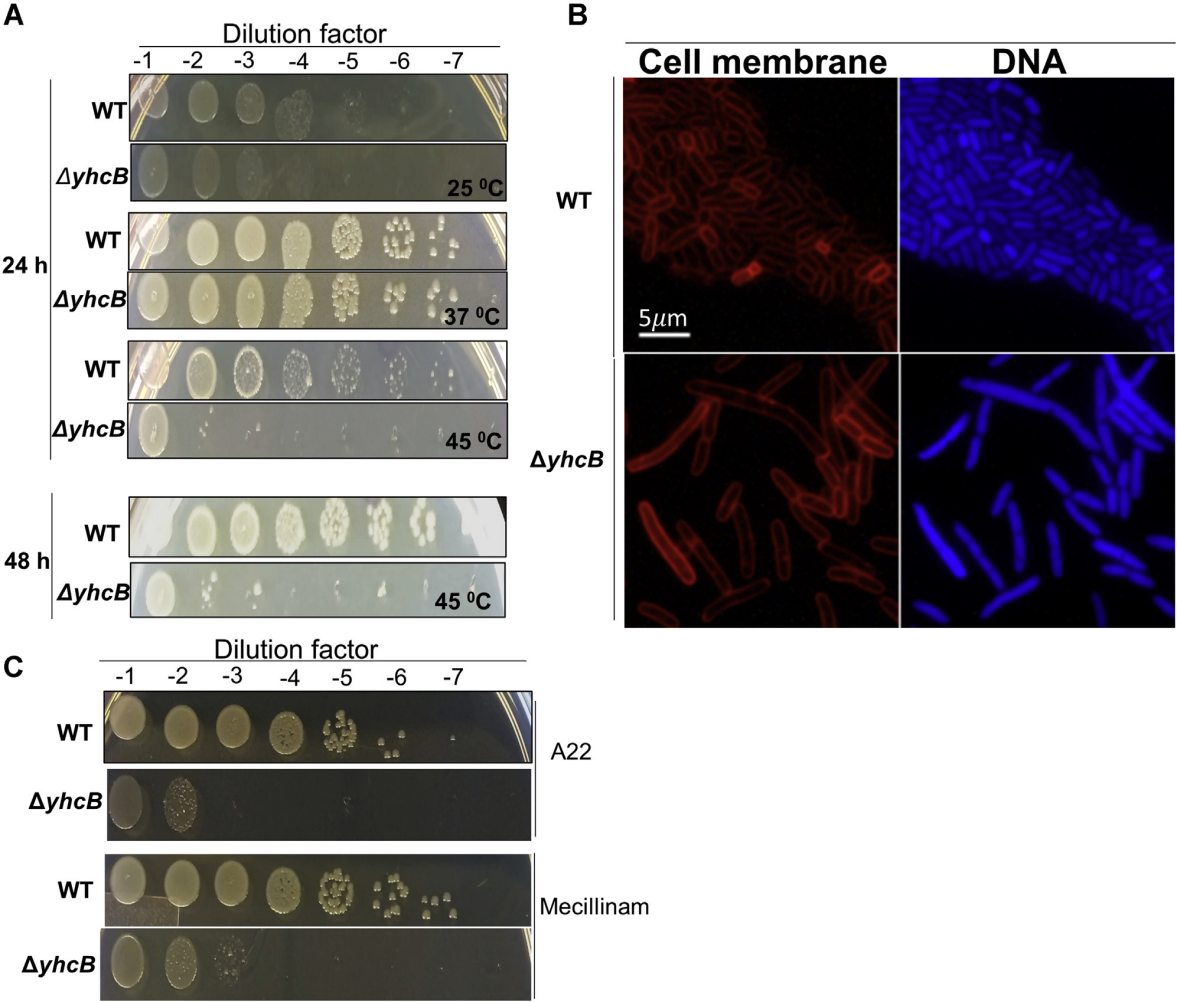
Δ***yhcB* deletion results in temperature sensitivity, filamentation, and susceptibility to antibiotics.***A*, temperature sensitivity of Δ*yhcB* cells, as shown by a serial dilution spot assay on LB/agar plates. The Δ*yhcB* cells are sensitive to high (45 °C) temperature after 24 h and 48 h. ***B***, micrographs of Δ*yhcB* cells in LB. The Δ*yhcB* cells were stained with FM4-64 and 4',6-diamidino-2-phenylindole dyes to visualize the cell membrane and DNA, respectively. Δ*yhcB* mutants resulted in elongated cells with and without septa. ***C***, susceptibility of Δ*yhcB* cells to cell wall–acting antibiotics. The Δ*yhcB* cells showed hypersensitivity toward cell wall–acting antibiotics in a serial dilution spot assay on LB/agar. *Top*: A22; *bottom*: Mecillinam.

To understand the function and phenotypes of *yhcB*, we used a *yhcB* deletion strain to carry out extensive phenotyping. The Δ*yhcB* strain grows with a mass doubling time of 25 min, whereas the WT doubles every 22 min. Morphologically, cultures of *E. coli* Δ*yhcB* exhibited increased cell lengths but reduced diameters (Fig. S2). The Δ*yhcB* cells grow normal under exponential (EXP) conditions but do not fully activate growth arrest toward the stationary (STAT) phase, which results in filaments. In STAT filamentous cells lacking *yhcB*, DNA segregation is often disturbed (Fig. 2*A*), in contrast to EXP cells, where DNA segregation appears to be normal (Fig. 2*B*). In addition, Δ*yhcB* cells showed several other phenotypes (*e.g.*, temperature sensitivity, defective cell envelope) (Fig. 3, *A* and *B*; Fig. S3*a–d*).

Notably, Sung *et al*. have provided additional evidence that support our results, including complementation of the aforementioned deletions by overexpression constructs, showing that the filamentation phenotype of their *yhcB* mutants were completely or significantly restored by YhcB expression (see Discussion for details).

### STAT phase cultures of Δ*yhcB* strain exhibit susceptibility to cell wall–targeting antibiotics

**Figure 4:**
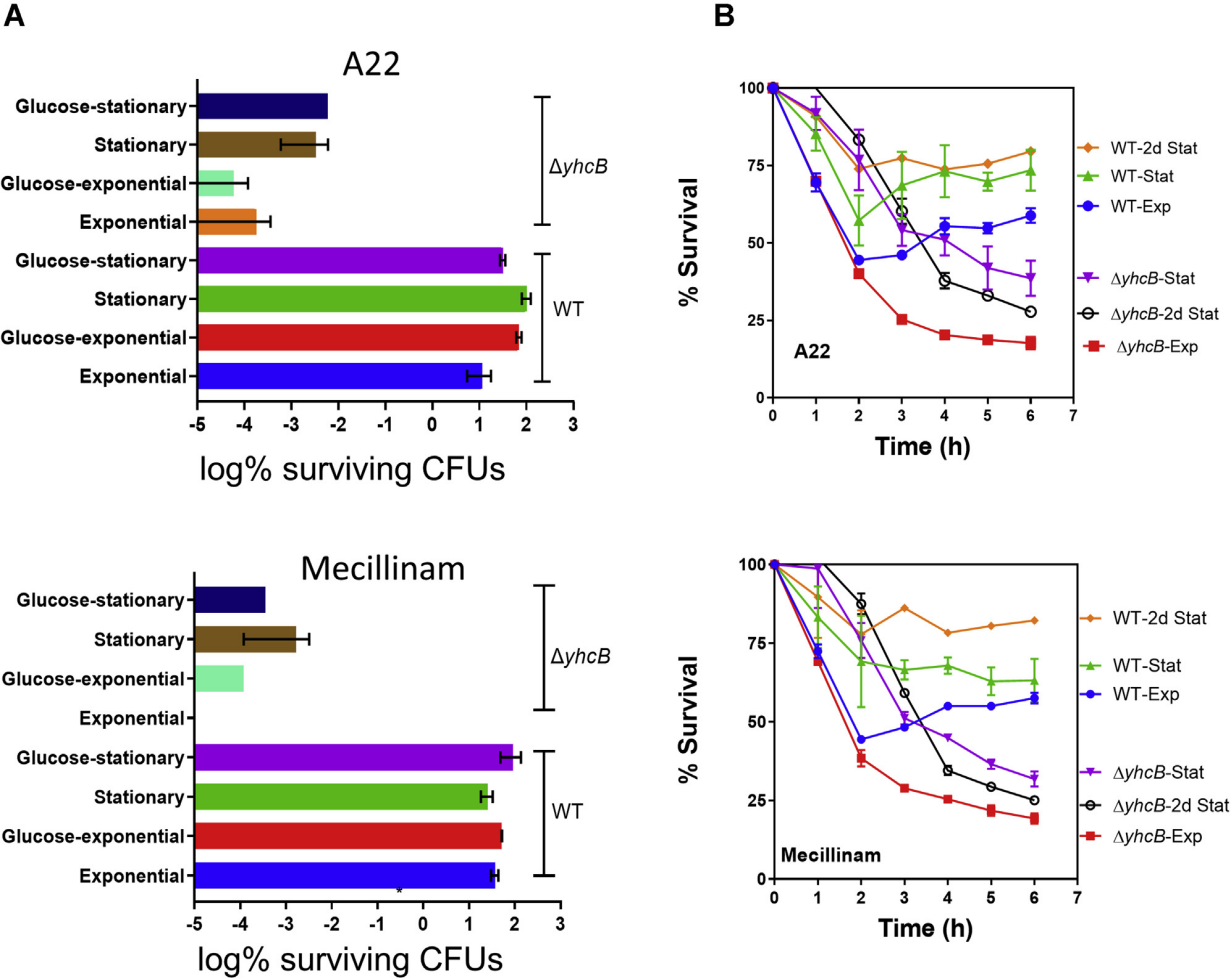
**Δ*yhcB* cells are hypersensitive to antibiotics**. A persister/survivor cell assay was used to measure survival of Δ*yhcB* cells upon antibiotic treatment for 6 h. *A*, fraction of surviving cells after exposure to antibiotics, expressed as log% CFUs. *B*, percent survival time course of exponentially growing (EXP) and stationary phase cells (STAT) in LB media (2d STAT represents 2-day-old stationary cells). *Top*: A22; *bottom*: Mecillinam.

Proteins of the cell elongasome such as MreB and PBP2 are direct targets of the cell wall antibiotics A22 and Mecillinam, respectively, and our experiments confirmed that the Δ*yhcB* strain was hypersensitive to both antibiotics (Fig. 3*C*). Most of the inhibitors/antibiotics that target cell envelope biogenesis, especially β-lactams, need actively growing cells to attain their maximum antibacterial activity. Given that a *yhcB* mutant strain exhibits hypersensitivity to antibiotics that target the bacterial cell wall (Figs. 3*C* and S4a (14)), we tested Δ*yhcB* cells in the early log phase, overnight (ON) and after 2 days in the STAT phase. We counted a lower number of survivor cells in Δ*yhcB* strain than in WT strain upon A22 treatment (Fig. 4*A*). No viable (persister) cell was observed after exposure of exponentially growing cells to Mecillinam (Fig. 4*A*). Two-day-old WT cells were least sensitive to cell wall–targeting antibiotics followed by ON and EXP cells. However, we observed the opposite trend for the Δ*yhcB* cells in terms of their sensitivity toward cell wall–acting antibiotics. All mutant cells were found to be hypersensitive to the cell wall antibiotics compared with ON cells (Fig. 4*B*). We also observed that WT cells adapted to antibiotic stress after 2 h, whereas Δ*yhcB* cells did not recover from the antibiotic shock even after 6 h (Fig. 4*B*). A22 and Mecillinam inhibited the growth of Δ*yhcB* mutants ∼3-fold more than WT cells after 6 h (Fig. 4*B*). The hypersensitivity of 2-day-old STAT cells indicates either an active peptidoglycan (PG) synthesis machinery in the STAT phase cells or defective cell envelope (Fig. S4*b*). The latter was also supported by the β-galactosidase assay that reports envelope leakiness (Fig. S3*c*).

### *yhcB* gene deletion leads to abnormal FtsZ ring and septum formation

**Figure 5:**
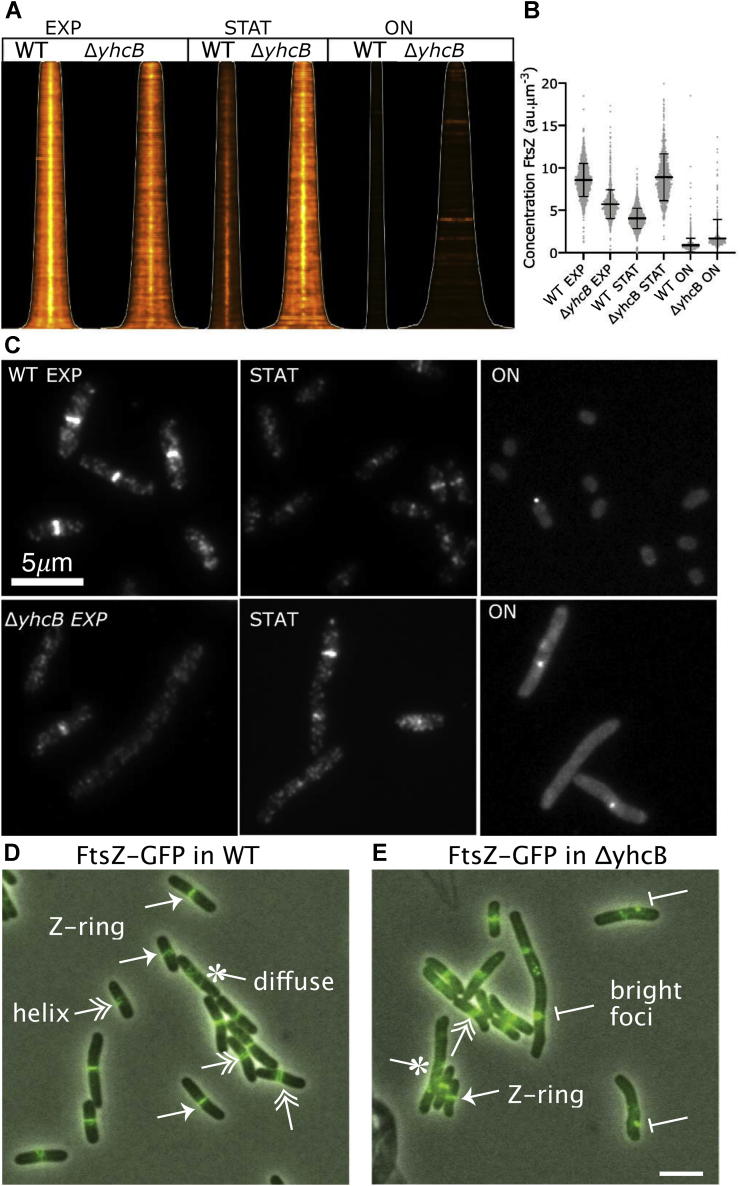
**Δ*yhcB* cells have abnormal FtsZ localization**. The Z-ring in Δ*yhcB* cells is not assembled properly as visualized by immunolabelling of FtsZ. *A*, the map of FtsZ fluorescence profiles sorted according to cell length. The *white line* indicates where the cell poles are. Brightness and contrast are the same for all profiles. B, the FtsZ concentration expressed in arbitrary units (“au”) of all the cells of each culture with the mean and SD indicated. Cells were analyzed in EXP, STAT, and ON phase (n = 1735, 1606, and 1321, respectively) for WT and Δ*yhcB* cells (n = 1154, 964, and 721, respectively). *C*, representative fluorescence images from all six cultures with WT (*top*) and Δ*yhcB* (*bottom*). The brightness and contrast of the images of the EXP and STAT cell is 0/13,000, whereas it is 0/1300 for the images from the ON cells. *D*, WT cells expressing FtsZ-GFP^sw^. *E*, the representative image of Δ*yhcB* cells expressing FtsZ-GFP^sw^. Different classes of FtsZ localization are indicated (*arrowhead* represents Z-ring, *double arrowhead* represents helix, *star* represents diffuse, *bar* represents bright foci). EXP, exponential; ON, overnight; STAT, stationary.

The aforementioned Δ*yhcB* phenotypes indicate defective cell division in Δ*yhcB* mutant cells. Therefore, to visualize the cell membrane and clearly discern septum formation, we stained the cells with SynaptoRedC2/FM4-64. No septum formation was observed in the majority of filamented cells (Fig. 3*B*). To determine if YhcB is necessary for successful formation of the bacterial divisome, we monitored FtsZ-ring formation in Δ*yhcB* cells. Immunolabeling with FtsZ-specific antibodies and secondary antibodies conjugated to a fluorophore in Δ*yhcB* cells showed that the Z-ring was not assembled properly/stably (Fig. 5, *A*–*C*). This was not due to degradation of FtsZ as its cellular concentration in Δ*yhcB* cells was sufficient to form FtsZ rings (Fig. 5*B*) and cells in all states potentially failed to form a Z ring. Notably, the Δ*yhcB* cells have more than twice the amount of FtsZ than the WT strain at the beginning of the STAT phase but that still did not rescue the phenotype. Furthermore, the FtsZ-ring formation appeared abnormal in the Δ*yhcB* strain with mislocalization of FtsZ (Fig. 5, *D* and *E*, Table 1).

**Table 1:** FtsZ localization in WT or Δ*yhcB* cells

Strain	Total cells	WT pattern	Diffuse	Abnormal
RM586 (WT)	675	88% ± 4.6	10% ± 0.41	2% ± 0.10
RM588 (Δ*yhcB*)	793	72.4% ± 3.4	15.4% ± 0.60	12.2% ± 0.50

WT pattern contains cells that showed a central Z-ring or helix. Diffuse indicates that cells did not show any discernible pattern of FtsZ localization. Abnormal indicates cells with bright foci, multiple Z-rings, or off-center Z-rings. Error is 90% confidence interval.

### Δ*yhcB* filaments have incomplete septa and impaired septal PG formation

**Figure 6:**
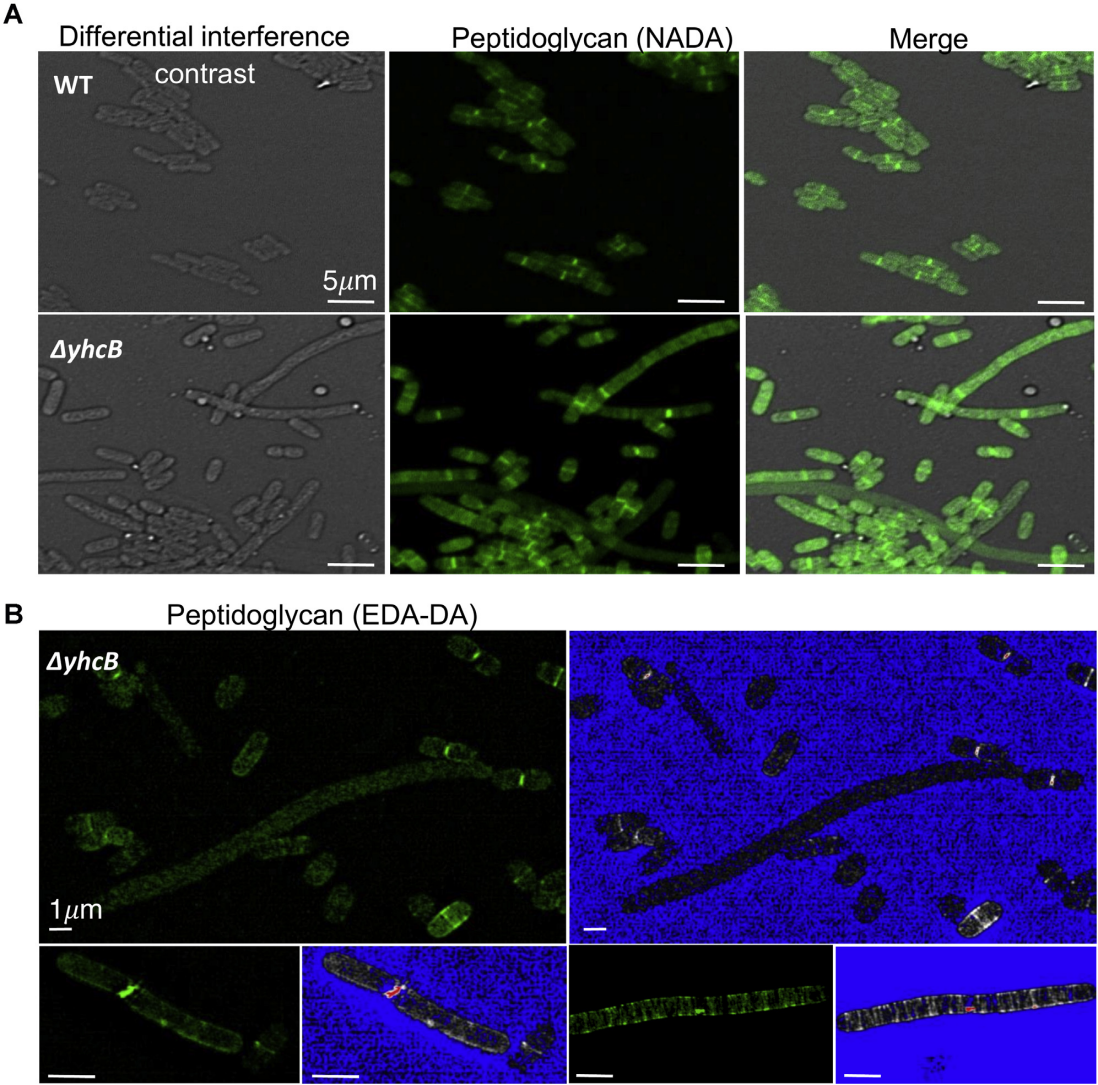
**YhcB affects peptidoglycan localization and septum formation**. *A*, WT *Escherichia coli* (BW25113) and Δ*yhcB* mutant cells subjected to a short (45 s) labeling pulse with the fluorescent D-alanine analog, NADA. Septa are observable within the smaller, more ‘WT’-looking Δ*yhcB* cells, whereas few visible septa are visible in elongated cells. *B*, structured illumination microscopy (SIM) of the Δ*yhcB* mutant strain labeled with the D-alanine dipeptide analog, EDA-DA. Long, filamentous morphotypes are shown that appear to lack probe incorporation (indicative of an absence of newly forming septa, *top panel*) or exhibit abnormal, ‘punctate’ labeling, similar to FtsZ labeling shown in *panel A*, (*bottom panels*). *Green* panels show images as they appear in the FITC channel, and *blue* panels show corresponding fluorescence intensity maps that range pixel intensities between 0 (*blue*) and 255.

The Δ*yhcB* strain showed impaired FtsZ ring formation, defective cell division, and hypersensitivity to antibiotics that target the cell wall (*e.g.*, PG synthesis). Therefore, to locate intracellular sites of YhcB activity, we monitored PG synthesis in Δ*yhcB* cells. PG labeling in a Δ*yhcB* strain was probed using a nontoxic, fluorescent D-amino acid analog of D-alanine (NADA) (15), which incorporates into the stem peptide of previously synthesized PG in living bacteria (Fig. 6*A*). In addition, we used another modified, D-amino acid dipeptide, ethynyl-D-alanyl-D-alanine (EDA-DA) (15, 16, 17), that incorporates specifically into the stem peptide of newly synthesized PG in the bacterial cytoplasm (Fig. 6*B*). Utilizing both probes, we observed far fewer labeled septa in the elongated forms of Δ*yhcB*. Similar to our previous observations, we also noticed a population of WT-like cells (in terms of the length and presence of labeled division septa). For the filamented forms, we observed what appeared to be septal labeling using both NADA and EDA-DA probes; however, septum formation often appeared either aberrant or incomplete (Fig. 6*B*). We did observe PG labeling around the cell periphery in some elongated cells, indicating that new PG synthesis by the elongasome appears to occur in these cells for some period of time. In conclusion, PG synthesis seemed to function apart from septum synthesis in filaments with diffuse Z-rings.

### YhcB genetically interacts with proteins of the cell division apparatus

**Figure 7:**
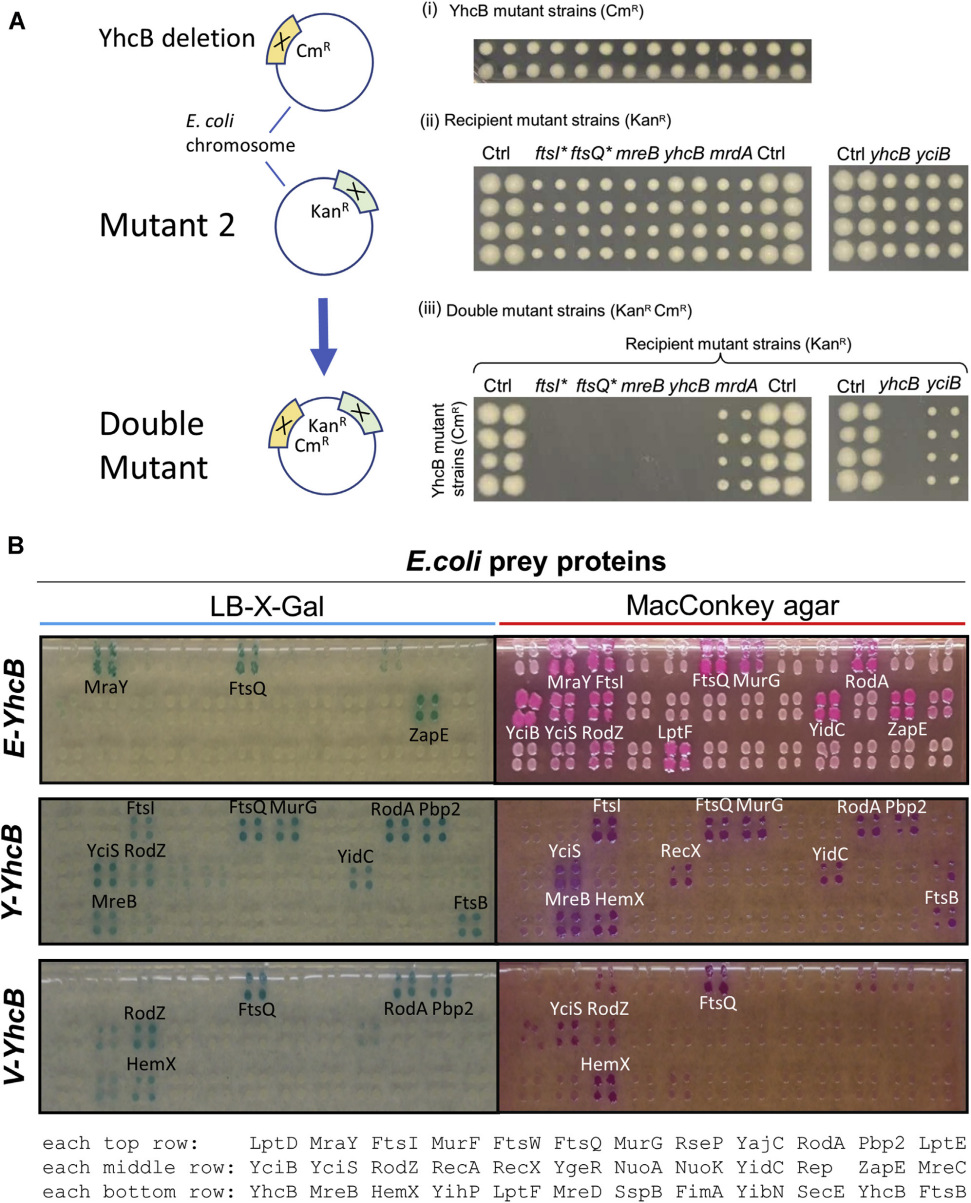
**Genetic and physical interactions of *yhcB*.***A*, double mutants of *yhcB* and *ftsI*, *ftsQ*, or *mreB*, respectively, show strong synthetic phenotypes, with less-severe effects in *yhcB-mrdA* and -*yciB* double mutants. Double mutants (iii) were generated in a rich medium by conjugating a *yhcB* deletion with the indicated F- recipient nonessential single-gene deletion or hypomorphic mutant strains (*asterisk*, when the gene is essential, (ii). Note that the “double-*yhcB*” mutant cannot grow in chloramphenicol + kanamycin media as it has only one of the two resistance genes. *B*, a representative bacterial two hybrid (B2H) screen of *Escherichia coli, Yersinia pestis*, and *Vibrio cholerae* YhcB bait proteins against *E. coli* prey proteins. The interacting proteins were screened by plating colonies on two different indicator media plates (LB/X-Gal, *left*, and MacConkey agar, *right*). The colored colonies showed positive interactions. See the text and Experimental procedures for details.

Given that *yhcB* is responsible for several phenotypes, we investigated the epistatic connections of *yhcB* with other bacterial genes (*i.e.*, if phenotypes of one mutation are modified by mutations in other genes). For that purpose, we used data from our previous envelope integrity study of *E. coli* screened under both auxotrophic (rich medium) and prototrophic (minimal medium) conditions. Strikingly, at a high stringent filtering of the genetic interaction score (|E-score|≥10; *p* ≤ 0.05; Table 2; Fig. 7*A*), we found 28 condition-dependent synthetic lethal interactions for gene pairs involved in cell division, cell shape, and cell wall biogenesis (or integrity), indicating that these genes are functionally related. Only the genetic interactions of *yhcB* with *ftsE* and *rodZ* were independent of the growth conditions and found in both media (Table 2).

**Table 2:** *yhcB* synthetic lethal genetic interaction pairs in cell division, cell shape, and cell wall biogenesis

Gene	E-RM	E-MM	Function
BcsB		S	Cell shape, glycan metabolism
CsrA		S	Cell shape
DacA		S	Cell wall biogenesis
DacB		S	Cell wall biogenesis
DacC		S	Cell wall biogenesis
DdpC		S	Cell wall biogenesis
DdpF		S	Cell wall biogenesis
**FtsA**		**S**	**Cell division, cell shape**
FtsE	S	S	Cell division
FtsK		S	Cell division
**FtsZ**		**S**	**Cell division, cell shape**
GlmU		S	Cell wall biogenesis
ManY	S		Cell wall biogenesis
MepA		S	Cell wall biogenesis
MipA		S	Cell wall biogenesis
MraY		S	Cell wall biogenesis
OppC	S		Peptide transport
OppD		S	Peptide transport
PgpB		S	Cell wall biogenesis
Prc	S		Cell division, cell wall biogenesis
PtsH	S		Sugar transport
PtsI	S		Sugar transport
**RodZ**	**S**	**S**	**Cell shape, cell wall biogenesis**
RsmG		S	rRNA processing
Slt		S	Cell division, cell wall biogenesis
YehU		S	Cell wall biogenesis
YfeW	S		Cell wall biogenesis
YgeR		S	Cell division
ZapB		**S**	**Cell division**
ZipA		S	Cell division, cell shape

E-ScoreRM (E-RM) (rich media) and E-MM (minimal media) indicate synthetic lethal genetic interactions, with “S” indicating strong synthetic lethal effects. See the text and Experimental procedures for details. Proteins with physical interactions are highlighted in bold (Table 3).

### YhcB copurifies with cell division proteins

Next, we searched for YhcB-interacting partners by expressing the protein with a C-terminal affinity tag from its native locus to maintain the physiological protein level. YhcB was then affinity-purified from detergent-solubilized cell extracts and analyzed by MS. In addition, we gathered proteins associated with YhcB in previous affinity-purified/MS and cofractionation studies (18), as well as from quantitative proteomics (19) without epitope tagging. By combining these four sets of data, we were able to identify 49 high-confidence proteins that copurified with YhcB and are involved in cell division/shape/biogenesis or maintaining membrane barrier function (Table S1). A subset of these were further explored (see below).

### Binary protein–protein interactions of YhcB

Based on the interactions we found for YhcB from the above proteomic screens, as well as their relevance to *yhcB* phenotypes (*e.g.*, RodZ), and results from other literature/database surveys, we chose 35 candidate proteins to test for direct interaction with YhcB (Table S1) using a bacterial two hybrid (B2H) system (20). We were able to verify a total of ten interactions in *E. coli* (Table 3) that were detected in multiple assays and/or conserved across species. Six of those were confirmed by the aforementioned MS-based proteomics dataset (Table S1), consistent with the validation rate typically observed for *E. coli* proteins using B2H assays (18, 21).

**Table 3:** Protein–protein interactions of YhcB in *Escherichia coli, Yersinia pestis*, and *Vibrio cholerae*, based on bacterial two hybrid screening (see Experimental procedures for details)

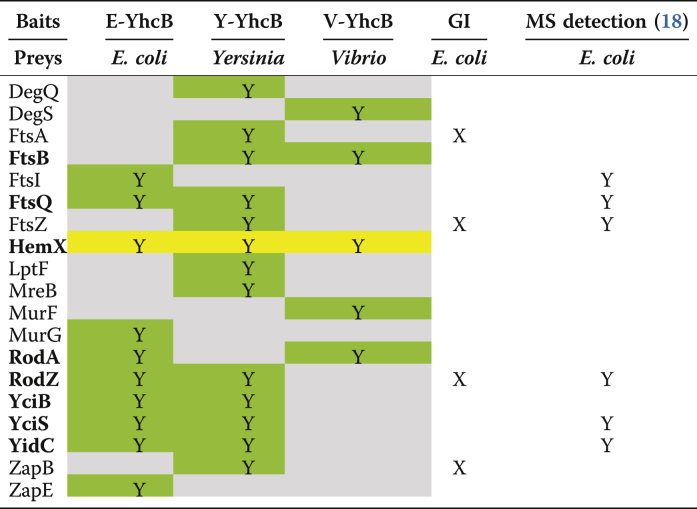

GI, genetic interaction.

Green boxes (Y) indicate positive interactions. The interaction with HemX (yellow) is conserved in all three species. See the text for details. For cross-species interactions, see Table S2.

To find biologically relevant and conserved interactions, we also tested the interactions found among *E. coli* proteins with their homologs from *Yersinia pestis* and *Vibrio cholerae* (Fig. 7*B*, see also Fig. 1). We detected 13 and five interactions of YhcB in *Y. pestis* and *V. cholerae*, respectively (Table 3). Six *Yersinia* and two of the *Vibrio* interactions were also detected in *E. coli* (Table 3). Interactions that were detected in at least two species were considered to be conserved (and thus as more reliable), and we found eight interactions in at least two species (Table 3). Only one interaction was detected in all three species, that of YhcB with HemX (Table 3).

We also tested cross-species interactions, that is, YhcB of *E. coli*, *Y. pestis*, and *V. cholerae* were tested against test proteins of *E. coli*, *Y*. *pestis*, and *V. cholerae* for both intraspecies and interspecies interactions (Table S2). For instance, three YhcB interactions were found between *E. coli* YhcB and *V. cholerae* RodA, ZapE, and HemX, respectively, although YhcB shares only 45% sequence identity with its orthologs in both species. In addition, six protein-protein interactions (PPIs) were found between *E. coli* and *Y*. *pestis*, which share 80% identity between their YhcB proteins (Table 3), and a few more across various combinations of the three bacteria (Table S2).

Importantly, YhcB interacts physically with proteins that comprise the cell elongasome (*e.g.*, RodZ, RodA) and divisome (*e.g.*, FtsI, FtsQ), complexes that are involved in cell wall biogenesis and septum formation. Consistent with this observation, in addition to a *rodZ* mutant, we were able to confirm synthetic lethal or loss of fitness interactions between *yhcB* and genes involved in cell division (*e.g.*, *ftsI*, *ftsQ*), cell wall biosynthesis (*mrdA*), and cell shape maintenance (*e.g*., *mreB*) (Fig. 7, *A* and *B*). These observations provide strong genetic and physical evidence that YhcB is involved in cell division and/or cell wall biogenesis.

### Crystal structure determination of the YhcB cytoplasmic domain

To reveal the molecular basis of YhcB function, we determined its crystal structure. Screening of several proteobacterial orthologs for their purification and crystallization behavior led us to focus on the structure determination of the cytoplasmic region of YhcB from the gamma-proteobacterium *H. ducreyi*, an opportunistic genital pathogen. We expressed a truncated version of 132 amino acid protein in *E. coli* with a deletion of the predicted N-terminal transmembrane (TM) α-helix (residues 2–30) (22). A hexahistidine affinity tag was added to its native C terminus for purification. We performed hydrodynamic analyses on the crystallization stock of this purified protein construct using size-exclusion chromatography with multiangle light scattering, which showed that it is primarily monomeric but forms small amounts of stable tetramer (2.5%) and hexadecamer (0.9%) in solutions (Fig. S5).

**Figure 8:**
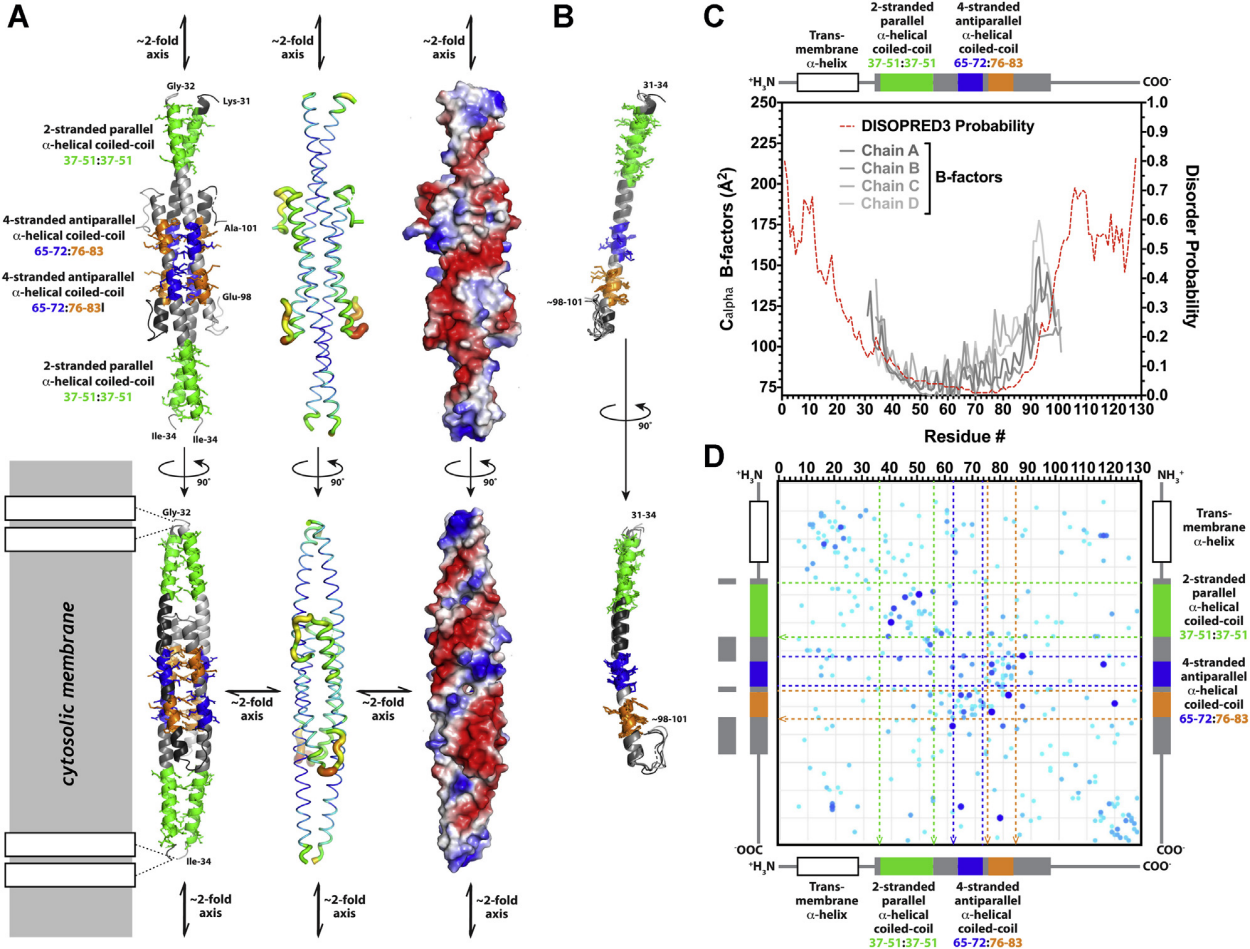
**Crystal structure of the YhcB ortholog from *Haemophilus ducreyi***. *A*, ribbon diagrams (*left*), B-factor-encoded backbone traces (*center*), and surface electrostatic representations of two views related by a 90° rotation around the long axis of the coiled-coil homotetramer in the asymmetric unit of the crystal structure (*right*). The *green* and *blue*/*orange* colors in the ribbon diagrams show, respectively, the segments participating in parallel and antiparallel coiled-coil interactions in the tetramer. The *rectangles* with *black borders* at *bottom left* schematize the approximate geometry of the predicted N-terminal transmembrane α-helix deleted from the crystallized construct. The *blue*/*narrow* to *red*/*wide* gradient in the B-factor-encoded backbone traces span 74 to 174 Å^2^. The fully saturated *blue*/*red* colors on the molecular surfaces encode vacuum electrostatic potentials of ± 93 kT calculated using the default parameters in PyMOL. *B*, ribbon diagrams showing least-square superposition of the four individual subunits in the asymmetric unit of the crystal structure, which are colored according to parallel *versus* antiparallel coiled-coil interaction as in the *leftmost* images in *panel A*. *C*, N and C termini of YhcB are predicted to be disordered. The backbone B-factors in the four subunits in the crystal structure (*gray* traces) plotted along with the probability of backbone disorder (*red* trace) calculated by the program DISOPRED3 (23) from the YhcB sequence profile. The secondary structure and parallel*/*antiparallel coiled-coil interactions observed in the crystal structure are schematized above the plot using the same color coding as in the *leftmost* images in *panel A*. *D*, evolutionary couplings predict intramolecular and intermolecular interactions in YhcB. Plot of pairwise evolutionary couplings (79) or probability of correlated evolutionary variations in the sequences of YhcB orthologs. The strength and statistical significance of each pairwise coupling are proportional to the diameter and darkness of the *blue* in the circles, which represent *p*-values from 0.58 to 1.0 (scaled scores from 1.0–2.7) calculated using ∼2.4 sequences per residue. The *largest* and *darkest blue dots* correspond to a coupling probability of 1.0, whereas the *medium blue dots* of intermediate size connecting residue 70 to residues 59 and 62 correspond to a coupling probability of ∼0.9. The *p*-values for the complete set of couplings shown are provided in the Supporting information.

This cytosolic region produced crystals that diffracted to ∼3 Å resolution, but they could not be solved using anomalous diffraction from selenomethionine-labeled protein because of the absence of any internal methionine residues in the native protein sequence. We therefore introduced I51M and L72M mutations at two conserved hydrophobic sites that have methionine in some YhcB orthologs, which enabled us to solve and refine the structure at 2.8 Å resolution using single-wavelength anomalous diffraction from selenomethionine-labeled protein (Table S3 and Fig. 8). Validation of the crystal structure is described in the Experimental procedures section.

The crystal structure of the cytosolic region of *H. ducreyi* YhcB shows a coiled-coil tetramer (Fig. 8*A*) in the asymmetric unit that is very likely to be a physiologically relevant assembly of the protein based on several lines of evidence described below. All four subunits form a long, continuous α-helix with an equivalent conformation (Fig. 8*B*) that starts at residues 34 to 37 and ends at residues 87 to 91 in the different subunits. At the C termini of these α-helices, the polypeptide chains could be traced into weak electron density through residues 98 to 101, but there is no interpretable electron density for the remaining 27 residues in any protomer. This entire segment of the protein has a high probability of backbone disorder according to the program DISOPRED3 (23), which predicts that over half of these disordered residues will participate in interprotein interactions. There is substantial amount of diffuse electron density in the crystal structure near the C termini of the protomers that cannot be modeled in any specific conformation but that presumably derives from this disordered protein segment. The inability to model this density accounts for the relatively high R factors of the refined structure (R_work_ = 30.8, R_free_ = 38.4). However, the other measures of refinement quality are all good (Table S3), and the close match between the refined backbone B-factors and the probability of backbone disorder according to Disopred3 (Fig. 8*C*) further supports the high quality of the refinement.

### The oligomeric assembly formed by YhcB

The core of the YhcB homotetramer is an antiparallel coiled-coil 4-helix bundle formed by residues 65 to 83 in each protomer (Fig. 8*A*). The interhelical packing pattern characteristic of coiled-coil interactions is interrupted by the alanine at position 73, which is responsible for the hole in the molecular surface visible in the view at the lower right in Figure 8*A*, but the register of the 4-helix antiparallel coiled-coil packing interactions between the helices is nonetheless continuous through this region. This tetramer represents a dimer of V-shaped dimers that make parallel coiled-coil packing interactions at their N termini spanning residues 37 to 51 (*i.e.*, the closed end of the V). The subunits in this dimer splay apart starting at glutamine 54, which enables the open ends of the V-shaped dimer to interact to form the antiparallel coiled-coil 4-helix bundle. The overall assembly thus combines parallel and antiparallel coiled-coil packing interactions to form a tetramer with 222 symmetry (*i.e.*, three orthogonal 2-fold axes that intersect at the center of the assembly in the hole in the antiparallel coiled-coil region formed by alanine 72). Although mixed parallel/antiparallel coiled-coil α-helical bundles have been observed before (*e.g.*, in PDB ID 4cq4 (24)), the program DALI (25) identifies the YhcB homotetramer as a novel protein structure because it has a unique tertiary structure in the region linking the parallel and antiparallel α-helical bundles.

The physiological relevance of this tetrameric assembly is supported by several lines of evidence, including strong evolutionary couplings (26) or pairwise evolutionary sequence correlations between the amino acids interacting in the central antiparallel coiled-coil bundle (Fig. 8*D*). The reliability of this computational analysis is supported by detection of the expected pattern of couplings between residues 3 to 4 apart in the long α-helix formed by each subunit that is observed in the crystal structure. The strongest cluster of intersubunit interactions in this analysis is between residues in the packing core of the antiparallel coiled-coil bundle, and couplings of this kind generally derive from direct physical contacts in a protein structure (27). Although *E. coli* YhcB was not found to self-associate in our B2H screens or in our copurification experiments, Li *et al.* (2012) did find a self-interaction in a B2H screen using a different construct geometry. Detection of productive B2H interactions can depend on the construct design because of the complexities of molecular geometry, especially for homo-oligomers, so evidence of self-association in some B2H data provides significant support for a physiological self-interaction. Finally, the program protein interfaces, surfaces and assemblies (28) also identifies the tetramer as a likely physiological oligomer based on quantitative analysis of its intersubunit packing interactions. Each subunit buries an average of 2530 Å^2^ of solvent-accessible surface area in interfaces in the tetramer (755 Å^2^ in the parallel coiled-coil interface and 790 Å^2^ and 988 Å^2^ in the antiparallel coiled-coil interfaces), which is in the range characteristic of physiological oligomers.

Although these observations all support the physiological significance of the tetramer observed in the crystal structure of *H. ducreyi* YhcB, the observation of a primarily monomeric structure in the crystallization stock suggests the affinity of the tetramer is such that it may reversibly dissociate *in vivo* dependent on the local concentration. The absence or presence of binding partners that have higher affinity for the tetramer than the monomer could also modulate tetramer formation *in vivo*. The failure to detect self-association in our copurification experiments is also consistent with relatively facile dissociation of the physiological tetramer.

Based on the location of its N-terminal TM α-helices, the YhcB tetramer is likely to sit like an ∼120 Å long handle parallel to the inner surface of the cytoplasmic membrane (CM) (lower left in Fig. 8*A*). The surface of this handle is characterized by a spiral pattern of strongly negative electrostatic potential (right in Fig. 8*A*) that is likely to influence YhcB's interprotein interactions and its interactions with the nearby negatively charged surface of the CM. This structure could serve as a reversibly forming assembly point for multiprotein complexes on the surface of the membrane dependent on the local concentration of YhcB.

### The interaction sites of YhcB are conserved

**Figure 9:**
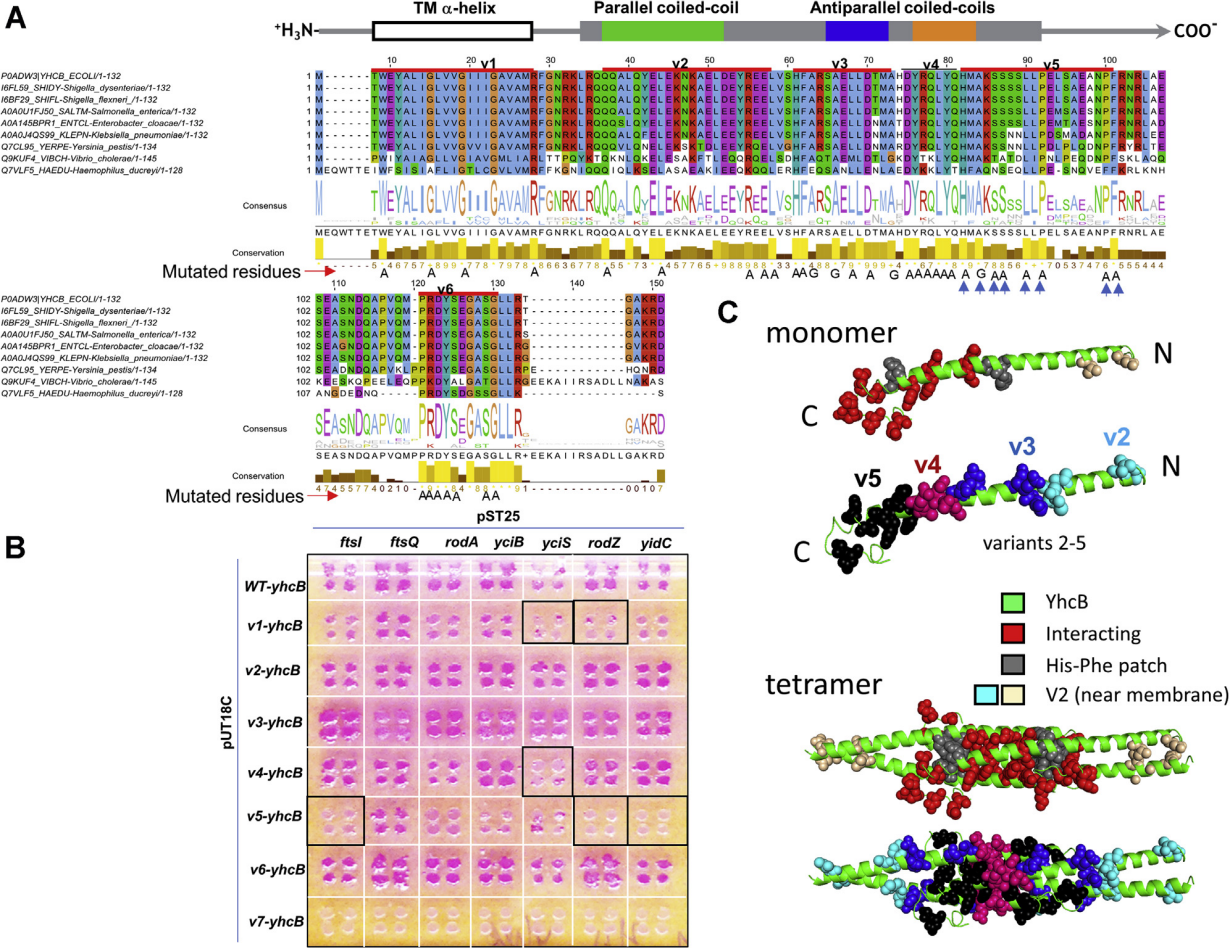
**Interaction sites on YhcB**. *A*, multiple sequence alignment of YhcB homologs across proteobacteria. The conserved residues are shown as a motif logo and histogram under the alignment, whereas the domain structure of *Haemophilus ducreyi* YhcB (Fig. 8) is shown above the alignment. The sequence is divided into six regions, v1 (N terminus) to v6 (C terminus), as indicated above the alignment. Highly conserved amino acid residues in each region were mutated as shown beneath the sequence. Each YhcB mutant (v1–v6) carries 4 to 6 substituted amino acid residues. *B*, bacterial two hybrid screens with YhcB mutants show the loss of specific interactions. The YhcB mutant (“variant”) v5 showed a loss of interactions with prey partners FtsI, RodZ, and YidC. The YhcB-v5 region possess several conserved residues predicted to be important for coiled-coil interactions as indicated by *arrows* underneath the sequence in panel A. No interactions were detected in the absence of the transmembrane (TM) region (v7). *C*, protein models show mutated and thus potentially interacting residues in both YhcB monomers and tetramers.

We used site-directed mutagenesis to map and identify the residues involved in PPIs of YhcB. We divided the *E. coli* YhcB protein into six different regions based on the conserved residues identified by multiple sequence alignment and Consurf-DB analysis (Fig. 9*A*). The resulting mutant variants cover different stretches of *yhcB* that we named v1 (N-terminal) to v6 (C-terminal). We also included a mutant lacking a TM region (v7, cytoplasmic domain only) to investigate what role membrane localization (or the TM region) plays in the proper functioning of YhcB (v7 had the N-terminal 21 amino acids deleted).

Only the conserved residues of these regions were mutated (Fig. 9*A*; mutated residues). Each YhcB variant had between four and eight amino acid substitutions, and all residues were replaced with either alanine or glycine. Each *yhcB* mutant variant was tested against the positive interacting partners identified previously in B2H screens. The amino acid substitutions of *yhcB* variants v1, v4, and v5 had the strongest effect on interactions and were thus considered as potential PPI sites of YhcB (Fig. 9*B*). Amino acids H76, A78, S80, S81, L84, P86, P94, and F95 of YhcB-v5 (shown as arrowheads in Fig. 9*A*) seem to form an interaction site for multiple interacting proteins, especially FtsI, RodZ, YciS, and YidC (Fig. 9*B*). YhcB-v1 includes the conserved residues in the TM region only. These residues seem to be required for interactions with YciS and RodZ. The rationale for substitution of TM residues was to test if the region had any effect on PPIs or whether it was only required for interactions with the membrane. Interestingly, the TM region is required for interactions with all proteins: when it is deleted, all interactions are lost (v7 in Fig. 9*B*, but see Discussion). Notably, the substitutions in *yhcB*-v3 appear to result in several stronger interactions (Fig. 9*B*). The locations of these mutations are indicated in the monomer and tetrameric models we derived from the structure (Fig. 9*C*).

## Discussion

### Phenotypes and interactions

In *E. coli, yhcB* is conditionally essential and required for survival at high temperatures, which is supported by previous large-scale screens (29, 30). Although the mechanisms underlying the temperature-related phenotypes remain unclear, heterologous expression of a *Caenorhabditis elegans* heat shock protein (CeHSP17) enabled *E. coli* cells to grow at 50 °C and was cross-linked and copurified with YhcB (31), linking YhcB to the *E. coli* heat shock response. Notably, we also observed an interaction between YhcB, YciS, and HemX proteins. YciS is a heat shock–induced protein (32) that has been copurified with YhcB and HemX (33).

In *E. coli* and *Salmonella*, YhcB expression was reduced significantly upon overexpression of SdsR, a small RNA transcribed by the general stress sigma factor σS (34, 35). It was proposed that SdsR-mediated *yhcB* repression may be the primary cause for the SdsR-driven cell lysis because of the perturbation of cell division. These authors have reported defective growth with filamented cells upon *yhcB* deletion (13, 35), which supports our results.

Sung *et al*. showed that *yhcB* deletions were restored by overexpressing YhcB protein, even when the TM segment was missing. Effective complementation excludes the possibility that the phenotype was caused by polar effects of the deletion mutants or independent mutations elsewhere in the genome. Although the phenotypes found by Sung *et al*., 2020, are similar to ours, most differences can likely be explained by somewhat different conditions and different strains (*E. coli* K-12 BW25113 in the Keio deletions used by us, but MG1655 used by Sung *et al*.).

### Envelope stress-related interactions

YhcB physically interacts with outer membrane stress sensor proteases (*degQ* and *degS*) (Table 3), and both YhcB and DegS were required for colonization of a host by *Vibrio* (36). Furthermore, both DegQ and DegS proteases are involved in protein quality control in the cell envelope (37), suggesting a role of *yhcB* in stress-related processes during cell wall biogenesis or in cell envelope integrity. Also, in *E. coli*, the *yhcB* gene is predicted to be a part of MazF regulon, and its mRNA is processed by MazF, a stress-induced endoribonuclease that is involved in post-transcriptional regulatory mechanism of protein synthesis globally in different stress conditions (38).

The hypersensitivity of Δ*yhcB* to cell wall–acting antibiotics (14), specifically to vancomycin, could be because of impaired cell wall biogenesis that leads to a permeable cell envelope (Fig. S3c) and is further supported by the involvement of *yhcB* as part of the secondary resistome against colistin, an antibiotic targeting the outer membrane, in *Klebsiella pneumoniae* (39).

### Role in cell division and/or envelope biogenesis

A functional cell envelope and PG biosynthesis is essential for cells to attach and form mature biofilms (40) and thus directly or indirectly explain *yhcB* cell wall–associated phenotypes. The hypersensitivity of Δ*yhcB* cells toward cell wall antibiotics in the STAT phase indicates an adaptive role during the STAT phase of bacterial cells. This notion is further supported by increased gene expression of YhcB during the STAT phase growth in *Salmonella* (34) and in *E. coli* (41) and the inability of Δ*yhcB* to reduce length growth during the STAT phase.

The PG labeling using NADA and ED-DA fluorescent probes that report on PG synthesis shows that lateral and septal PG synthesis is functioning globally as in WT cells, apart from the positions of diffuse Z-ring localization. This suggests that YhcB is likely not directly involved in PG synthesis. However, a synthetic lethal and a physical interaction was observed between YhcB and YciB (Fig. 7, *A* and *B*), a protein previously shown to be involved in PG synthesis (42) and a predicted intracellular septation protein (43). The deletion of *yhcB* does result in not only filamentation but also diffuse localization of Z-rings in those filamented cells. These phenotypes, together with the genetic and physical interactions of YhcB with FtsI, FtsQ, FtsZ, RodA, RodZ, and MreB, strongly support its role in cell division.

**Figure 10:**
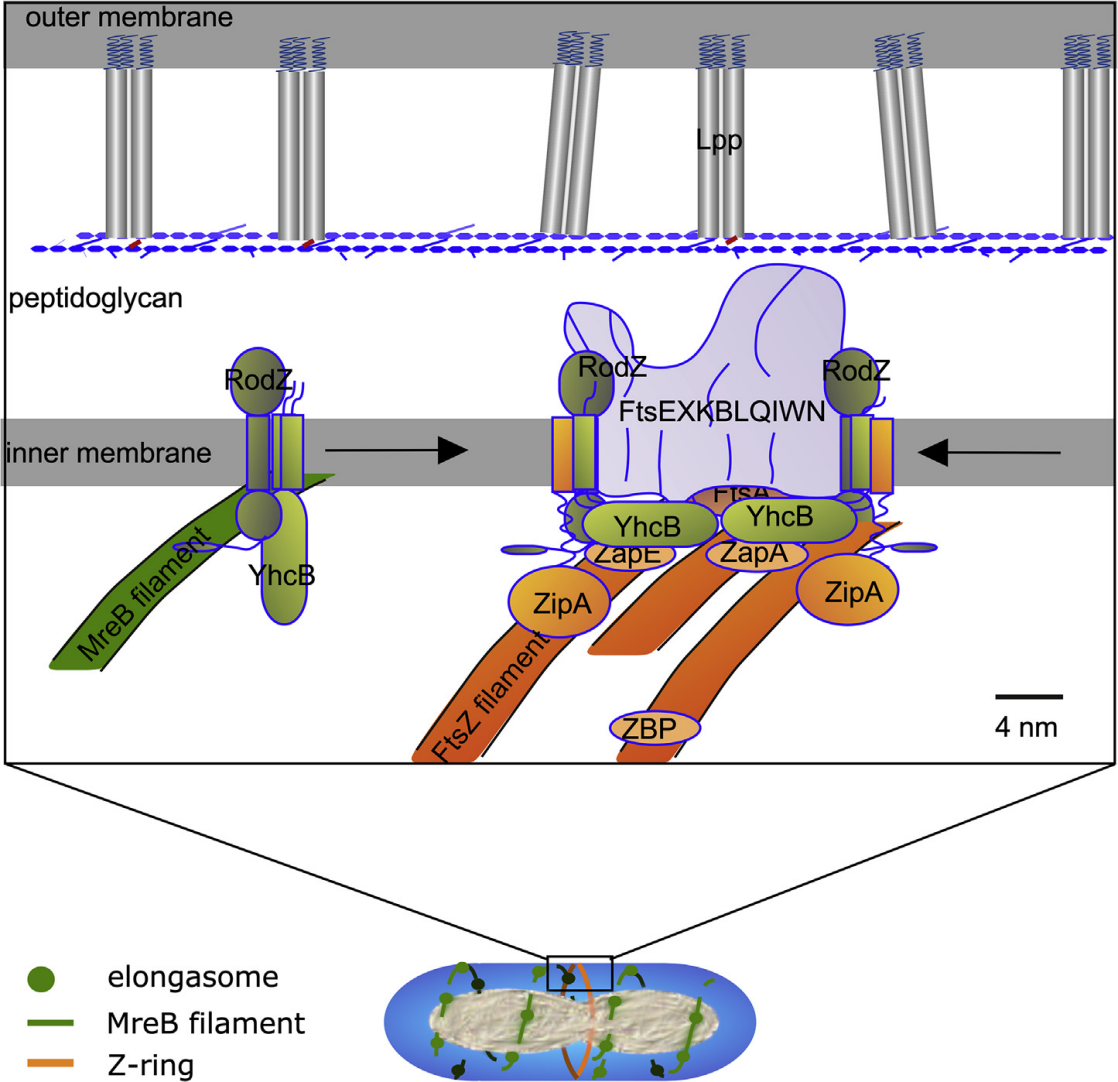
**Model for YhcB function in cell division and Z-ring width maintenance.** YhcB interacts as a dimer with RodZ that is part of the elongasome (schematic below). During peptidoglycan synthesis, MreB (*green* filament) moves perpendicular to the length axis underneath the cytoplasmic membrane. Some of these filaments close to midcell will be stalled by the Z-ring in the nascent state (*orange*). While some of the elongasome proteins will be involved in preseptal peptidoglycan synthesis on the periplasmic side of the cytoplasmic membrane, RodZ and YhcB interact with FtsZ filaments. As YhcB is likely present on both sides of the Z-ring, the two dimers can associate into the tetrameric complex as observed by crystallography. This produces a bridge of ±12 nm that can have multiple interactions with divisome proteins (here combined in one *gray* structure, “FtsEXKBLQIWN”) as observed by BTH. The Z-ring is formed by many filaments (with about 20 subunits each) that are connected by various FtsZ binding protein (ZBP, ZapA, and ZapE) and linked to the cytoplasmic membrane by FtsA and ZipA (and possibly YhcB). With an average width of about 10 nm, the Z-ring is of the similar size as the RodZ–YhcB complex.

To accommodate our own and other observations, we propose a model for YhcB's role in cell division, which is based on previous models (44) (Fig. 10). YhcB interacts with several division proteins, including RodZ and RodA, suggesting that it is involved in the elongasome. Midcell localization of RodZ was shown to be essential for Z-ring formation (45). RodA forms a permanent complex with PBP2 (46), which was shown to be initially present at midcell during Z-ring formation (47). The combined interactions of YhcB suggests that the elongasome brings YhcB to the assembly site of the divisome during preseptal PG synthesis. The divisome is a highly dynamic complex; hence, its isolation has been only partly successful (47), but YhcB was detected as one of the divisome proteins isolated from cells in the EXP and STAT phases using MS (47).

Consequently, many proteins have been reported that are supposed to help the FtsZ filaments to interact with each other (48, 49). But how are the boundaries of the Z-ring constrained? On the periplasmic side of the CM, preseptal PG synthesis is thought to provide the borders in between which the new septum should be synthesized (50, 51). We suggest that YhcB helps provide this function on the cytoplasmic side of the CM. While associated with RodZ at elongasome positions, YhcB may be dimeric or monomeric, but these interactions are dynamic and likely transient. When the elongasome is stalled at the nascent Z-ring from both sides of the ring, YhcB could come sufficiently close to form a weakly interacting tetramer parallel to the surface of the cytoplasm. This would provide a restricted width of the Z-ring of about 120 Å, which correlates well with the average width of the Z-ring of ±115 Å (52) and suggests that YhcB helps determine the width of the Z-ring. The surface of the coiled coil of YhcB is sufficiently charged to interact with the membrane and a number of cell division proteins and may tether the assembly in close proximity of the CM.

### Implications of the structure for biochemical function

The crystal structure of the *H. ducreyi* ortholog (Fig. 8) shows that its interaction sites cluster near the antiparallel alpha-helical coiled coil at the center of the YhcB tetramer (Fig. 9*C*). Therefore, when the local concentration of YhcB is sufficient to drive homotetramerization, the tetramer and its 222 symmetry will enable it to mediate specific interactions tethered directly to the inner surface of the CM. These interactions could serve as a focal point for organization of supramolecular complexes controlling membrane morphology and dynamics during cell division. At lower concentrations, the monomer of YhcB could alternatively sequester the interaction interfaces of binding partners in a dissociated state to drive membrane morphology and dynamics in a different direction. The data presented in this article support YhcB playing a role in envelope biogenesis/integrity and cell division in gamma-proteobacteria. Biophysical studies of the interacting complexes identified in this article, including cryo-EM reconstructions of the membrane-bound complexes and cocrystallization experiments, should provide deeper and more specific insight into the details of the related molecular mechanisms. Finally, we conclude that the function of YhcB is provided by unrelated proteins or other divisome proteins; hence, we predict that a more detailed comparative genomic analysis will reveal functionally similar proteins in species that do not have YhcB.

Given its function as determined here, we propose to rename YhcB to ZapG (Z-ring associated protein G), in analogy to ZapA, ZapB, ZapC, and ZapD (53).

## Experimental procedures

### Bacterial strains and reagents

All strains used are listed below in their context of use. Strains were grown in LB media at 37 °C unless otherwise mentioned. The KOs were obtained from the *E. coli* Keio collection (54). PCR was used to confirm the *E. coli* Keio KOs using gene-specific primers. *E. coli* TOP10 and DH5α were used for cloning. For protein expression, *E. coli* BL21(pLys) cells were used. *E. coli* was selected at 100 μg/ml ampicillin and/or 35 μg/ml chloramphenicol for expression in liquid media. All the expression experiments were performed at 30 °C unless otherwise mentioned. Antibiotics A22 and Mecillinam were purchased from Sigma-Aldrich (now Millipore Sigma).

### Phylogenetic analysis and comparative genomic analysis

To determine potential for conservation of genes coding for our proteins of interest across bacterial species, we used the following methods. Starting with each gene's UniProtKB identifier for *E. coli* K12, we identified membership of each in an orthologous group (OG) as defined by EggNOG v5.0 (55). Gene names, UniProtKB IDs, and corresponding EggNOG OGs are as follows: *ftsI* (P0AD68, COG0768), *ftsQ* (P06136, COG1589), *ftsZ* (P0A9A6, COG0206), *rodA* (P0ABG7, COG0772), *rodZ* (P27434, COG1426), *yciS* (P0ACV4, COG3771), *yhcB* (P0ADW3, COG3105), and *yidC* (P25714, COG0706). In each case, the OG based on the broadest taxonomic definition was used (*i.e.*, a COG). We then assembled a tree of 197 bacterial species and strains based on their NCBI taxonomy (56) and, for each, determined the presence of at least one gene with membership in each of the above OGs as per EggNOG. The presence of these OG members was mapped and visualized with the iTOL tool v4 (57).

Genomic colocalization analysis was performed using the SEED annotation environment across representative members of sequenced bacterial species (58).

### Gateway cloning

Gateway cloning was performed according to instructions provided by the manufacturer (Invitrogen). The ORFs as entry clones for test proteins were obtained from the *E. coli* ORFeome clones assembled into the pDONR221 vector system (59). Then, the attL-flanked ORFs were cloned into the Gateway-compatible, attR-flanked bacterial two-hybrid (BACTH)-DEST plasmids (pST25-DEST, pUT18C-DEST, and pUTM18-DEST) using the LR reaction to generate attB-flanked ORFs in expression vectors. The plasmid preparations were performed using NucleoSpin column kits (MACHEREY-NAGEL). For the details of the B2H vectors and protocol, please refer to (60, 61).

### B2H screening

B2H screens were carried out as described previously (61). Briefly, the expression constructs of test proteins encoding the T25-X and T18-Y fusions were cotransformed into an adenylate cyclase–deficient *E. coli* strain (BTH101). The competent cells were prepared using standard protocols (62). The cotransformants were selected on LB plates containing 100 μg/ml ampicillin and 100 μg/ml spectinomycin at 30 °C after 48 h. The selected cotransformants were screened on indicator plates at 30 °C for 36 to 48 h. The positive interactions were detected by specific phenotypes on indicator plates, that is, blue colonies on LB–X-Gal–IPTG or red on MacConkey–Maltose medium. For quantification of PPIs (where required), the β-galactosidase assay was used (63). The details of test proteins are shown in Table S4.

### Affinity purification combined with MS and genetic crosses

YhcB fused to sequential peptide affinity tag, chromosomally at the C terminus, was confirmed by immunoblotting using anti-FLAG antibody and then purified in the presence and absence of various mild nonionic detergents, essentially as described (18). The stably associated proteins were detected by MS using the SEQUEST/STATQUEST algorithm, following established procedures (18, 33).

Genetic crosses were conducted as previously described (33) by conjugating Hfr Cavalli (Hfr C) *yh*c*B::Cm*^*R*^ donor gene deletion mutant marked with chloramphenicol against the select set of F- ‘recipient’ nonessential single gene deletion or essential hypomorphic mutants marked with kanamycin resistance, including functionally unrelated gene *JW5028* (64) from the Keio single-gene deletion mutant library, to generate digenic mutants after both antibiotic selection.

### Mapping protein–protein interaction site(s): mutagenesis of *yhcB*

To map interaction site(s), YhcB was divided into six different regions, and in each region, 3 to 4 site-specific substitutions were inserted. Also, a cytoplasmic version without the TM region of YhcB was constructed. Only conserved residues of YhcB were mutated (as shown in Fig. 9*A*). Mutant DNA sequences encoding specific mutants were synthesized as full gene sequences by GeneArt (Thermo Fisher pvt Ltd). These sequences were further cloned into pDNOR/Zeo using the BP Clonase reaction of Gateway cloning (Invitrogen). The transformants with correct sequences were confirmed by sequencing at least two different clones. The ORFs were further subcloned into B2H vector pUT18C followed by cotransformation and screening for interactions against prey proteins as discussed above (see B2H screening).

### Growth inhibition/sensitivity against drugs

The growth of both WT and Δ*yhcB* strains was monitored in different media and in different conditions, such as different carbon sources, antibiotics, as well as rich and selective media, each in 96-well microplates at 37 °C. The bacterial growth was measured as the absorbance at 562 nm using a plate reader. The percent inhibition (or survival) was calculated as previously described (65).

### Antibiotic susceptibility testing (serial dilution assay)

An ON culture of *E. coli* strains (both WT and Δ*yhcB*) was tested for susceptibility toward cell wall antibiotics using serial dilutions. About 10^7^ cells/ml were serially diluted, and 5 μl of each dilution was spotted on LB with or without added antibiotic or other compounds (*e.g.*, 1% carbon sources). For MacConkey plates, 3 μl of each dilution was used. The plates were then imaged after 24 h or at other specific time points (see the text for details). A22 (1 μg/ml) or Mecillinam (0.12–0.25 μg/ml) was used in dilution assays on hard agar media. These concentrations were chosen based on effective ranges tested by Nichols *et al*. (14) (0.5, 2, 5, and 15 μg/ml for A22, resulting in [log] reductions of growth by −1.015628, −4.344713, −3.311473, and −3.978085, respectively), and Mecillinam (0.03, 0.06, 0.09, and 0.12 μg/ml, resulting in [log] reductions of −0.339263, −4.244134, −8.923793, and −6.08356, respectively).

### Persister/survivor cell assay

Persister/survivor cell assays were performed as reported previously (66). Persistence was determined by determining the number of CFUs upon exposure to A22 (1 μg/ml) and Mecillinam (0.12 μg/ml). We determined the number of persister/survivor cells in the Δ*yhcB* strain upon exposure to cell wall antibiotics for 6 h. The ON culture was subcultured at 37 °C for 2 h, and the cells in the early log phase were treated with antibiotics. The ON cells were used as STAT phase cells. For determination of CFUs, 2 μl of culture (10^7^ cells/ml) was resuspended in a fresh medium, serially diluted, and plated on solid LB medium. The number of survivor cells was determined as CFUs upon antibiotic treatment. The CFUs were expressed as percent survival of treated *versus* untreated cells.

### FtsZ localization

The FtsZ ring formation and localization was monitored using both immunolabeling and GFP fusion of FtsZ. The Δ*yhcB* and its parental strain BW25113 (WT) were grown in LB at 37 °C for 24 h (ON) and then diluted 1:1000 and grown to an absorbance at 650 nm of 0.3 (EXP) or to an absorbance at 650 nm of 1.2 (STAT), fixed for 15 min by addition of a mixture of formaldehyde (final concentration 2.8%) and glutaraldehyde (final concentration 0.04%) to the cultures in the shaking water bath and immunolabeled (67) with Rabbit polyclonal antibodies against FtsZ as described previously (68). As the secondary antibody, donkey anti-rabbit conjugated to Cy3 or to Alexa488 (Jackson ImmunoResearch) diluted 1:300 in the blocking buffer (0.5% (wt/vol) blocking reagents (Boehringer, Mannheim, Germany) in PBS) was used, and the samples were incubated for 30 min at 37 °C. For immunolocalization, cells were immobilized on 1% agarose in water slabs coated object glasses as described (68) and photographed with an ORCA-Flash 4.0 (Hamamatsu, Japan) charge-coupled device camera mounted on an Olympus BX-60 fluorescence microscope through a 100×/numerical aperture 1.35 oil objective. Images were taken using the program ImageJ with Micro-Manager (https://www.micro-manager.org). Phase contrast and fluorescence images were combined into hyperstacks using ImageJ (http://imagej.nih.gov/ij/), and these were linked to the project file of Coli-Inspector running in combination with the plugin ObjectJ (https://sils.fnwi.uva.nl/bcb/objectj/). The images were scaled to 15.28 pixel per μm. The fluorescence background has been subtracted using the modal values from the fluorescence images before analysis. Slight misalignment of fluorescence with respect to the cell contours as found in phase contrast was corrected using Fast-Fourier techniques as described (68). The length, diameter, and fluorescence concentration were measured using Coli-Inspector running in combination with the plugin ObjectJ (https://sils.fnwi.uva.nl/bcb/objectj/) as described (68).

For GFP-tagged FtsZ localization, the cells were grown at 37 °C in LB media to the EXP phase. Imaging was performed on M16 glucose plus casamino acids pads with 1% agarose at room temperature. Phase-contrast images were collected on a Nikon Eclipse Ni-E epifluorescent microscope equipped with a 100×/1.45 NA objective (Nikon), Zyla 4.2 plus camera, NIS Elements software (Nikon). A functional FtsZ fusion was made by inserting msfGFP at an internal site of FtsZ and replacing the native copy of FtsZ with the fusion protein (69).

### PG labeling and localization

The PG labeling studies were conducted as previously reported (15, 16). Briefly, ON cultures were started from single colonies grown from −80 °C freezer stocks (plated ON). Experimental cultures were then started in 5 ml of LB. Double the amount of the WT strain was used to inoculate cultures for the *yhcB* mutant (50 μl *versus* 100 μl in 5 ml) to attain absorbance as close as possible after two and a half hours of growth (absorbance at 600 nm of 0.8 and 0.7, respectively). This was performed to minimize the time required to back-dilute and achieve exactly equivalent absorbance readings, which likely would have had an effect on the rate of PG synthesis/and turnover.

We took logarithmic growing cultures (WT in LB and Δ*yhcB* in LB + 1% glucose) and conducted a short pulse with our first gen probes (NADA) second gen probes (EDA-DA) for 45 s. Glucose supplementation was utilized in the Δ*yhcB* culture to ensure each strain achieved comparable growth kinetics. After the short pulse, bacteria cultures were fixed immediately in 70% (final concentration) ice-cold ethanol for 20 min. NADA-labeled cells were washed three times in PBS, mounted on 1% agar pads, and imaged *via* a Zeiss 710 confocal laser scanning microscope. EDA-DA-labeled cells were subsequently bound to azide-conjugated Alexa Fluor 488 *via* a click chemistry reaction using a Click-iT Cell Reaction Buffer Kit (Invitrogen), as previously described (16). Cells were then washed three times in PBS +3% BSA, once in PBS, mounted on 1% agar pads, and imaged *via* Zeiss Elyra PS1 super resolution microscope in the structured illumination mode. Images are representative of 20 fields of view observed per condition/strain examined.

### Light microscopy and image analysis

The cells were stained and imaged to visualize cell membrane and nucleoid using FM4-64 SynaptoRed C2 (FM4-64 (4-[6-[4-(diethylamino) phenyl]-1,3,5-hexatrien-1-yl]-1-[3-(triethylammonio) propyl] pyridinium dibromide, Biotium Inc) and 4',6-diamidino-2-phenylindole, respectively. The cells were imaged on an Olympus BX41 microscope at 100× in a dark room. Images were captured with a microscope digital camera (AmScope MU1400). ImageJ software was used for measuring cell dimensions/length (70).

### Protein expression, purification, and light-scattering analysis

Residues 31 to 128 from the YhcB ortholog in *H. ducreyi* (HD1495, UniProt ID Q7VLF5, Northeast Structural Genomics Consortium target HdR25) were cloned into a pET21-derived T7 expression vector between an N-terminal initiator methionine residue and a C-terminal affinity tag with sequence LEHHHHHH, and this vector was deposited at the ASU Biodesign Institute (http://dnasu.org/DNASU/GetCloneDetail.do?cloneid=338479). Cloning, purification, and quality control analysis methods were described previously (71). In brief, after growing cells to a logarithmic phase at 37 °C in chemically defined MJ9 medium with 0.4% (w/v) glucose, protein expression was induced ON at 18 °C with 1 mM IPTG. Soluble protein was purified by Ni-NTA chromatography followed by Superdex 75 gel filtration in 100 mM NaCl, 5 mM DTT, 20 mM Tris HCL, pH 7.5. Pooled fractions were ultrafiltered in an Amicon device before flash-freezing in liquid N_2_ in single-use aliquots at crystallization concentration. Protein quality was characterized using SDS-PAGE, MALDI-TOF MS (12574.8 Da observed *versus* 12549.6 predicted for selenomethionine-labeled WT protein), and size exclusion chromatography/multiangle light scattering in the gel-filtration buffer using a Shodex KW802.5 column (Showa Denko) with a Wyatt Technology detector system (Fig. S5).

### Protein crystallization, X-ray structure determination, and refinement

Crystallization screening and optimization were performed using the microbatch method under paraffin oil (72, 73). After optimization, YhcB crystals useful for structure determination were grown in drops composed of 1.0 μl of protein and 1.0 μl of precipitant solution (2.0 M ammonium sulfate) under paraffin oil (Hampton Research). Crystals were cryoprotected by brief passage through the precipitant solution plus 20% (v/v) ethylene glycol before mounting. The structure was solved using single-wavelength anomalous diffraction phasing (74) of a selenomethionine-labeled construct harboring I51M and L72M mutations, which crystallized similarly to the WT construct. These mutations were introduced to increase selenomethionine phasing power compared with the WT construct, which only has a single N-terminal methionine that is disordered in the crystal structure. The mutations were introduced at uniformly hydrophobic positions that show methionine in some orthologs in a YhcB sequence alignment, based on the premise that such positions are likely to be at least partially buried and therefore well ordered and provide good phasing power. Diffraction data were collected at 100˚K on beamline 19-ID at the Advanced Photon Source using X-rays at the Se K-edge (λ = 0.979 Å) and processed using HKL2000 (75). The structure was solved and refined at 2.8 Å resolution using PHENIX (76), built using interactive cycles in Coot (77), validated using PROCHECK (78), and deposited in the research Collaboratory for structural bioinformatics Protein Data Bank under accession code 6UN9. Data collection and refinement statistics are shown in Table S3.

The relatively high free R-factor for a structure at this resolution (38.4%) is attributable to the low mean intensity of the diffraction dataset (<I/σ_I_> = 4.1>) combined with the high degree of disorder in the crystallized construct (Table S3). Over 30% of residues are disordered and could not be modeled at all, while greater than 10% of the residues are only partially ordered, preventing accurate modeling with a single coordinate model with individual atomic B-factors. The disordered residues and the refined B-factors of the modeled residues (Fig. 8*C*) both correlate very closely with the probability of backbone disorder calculated by the program DISOPRED3 (23), which uses exclusively primary sequence data and is therefore completely independent of the crystal structure. Furthermore, the accuracy of the structure solution and refined coordinate model are supported by four additional factors, all of which are independent of one another and the backbone disorder prediction. First, the interprotomer contacts in the structure correlate strongly with pairwise evolutionary couplings in the YhcB protein family (Fig. 8*D*) as calculated by the program GREMLIN (79), which also uses exclusively primary sequence data and is completely independent the crystal structure. Second, an anomalous difference Fourier map calculated with the refined phases shows strong peaks at the positions of the selenium atoms in the engineered selenomethionine residues in the protein construct and no significant peaks anywhere else in the unit cell (Fig. S6*A*). Third, the 2f_0_-f_c_ electron density map calculated from the refined coordinate model shows excellent agreement with the model consistent with the 2.8 Å overall resolution of the crystal structure (Fig. S6*B*). Finally, the crystallographically related tetramers fill the unit cell and make appropriate packing interactions to stabilize the modeled structure in the lattice, which has a 65% solvent content (Fig. S6).

### Protein structure analysis

Coiled-coil sequence propensity was analyzed using the program Coils (80), which indicates high probability of coiled-coil formation for residues 44 to 64, 37 to 75, and 30 to 82 for windows of 14, 21, and 28 residues, respectively. Coiled-coil packing interactions in the crystal structure were analyzed using Socket (81) and Twister (82). Buried solvent-accessible surface area was calculated using protein interfaces, surfaces and assemblies (28). Backbone disorder probability was calculated using DISOPRED3 (23), and evolutionary couplings were calculated using Gremlin (79). Molecular graphics images were generated using PyMOL (https://pymol.org/2/), which was also used to calculate *in vacuo* surface electrostatics.

## Data availability

YhcB structure data presented here have been deposited in the RCSB Protein Data Bank (PDB) under accession code 6UN9. All remaining data are contained within the article.

## Supporting information

This article contains supporting information (7, 83, 84, 85).

## Conflict of interest

G. T. M. is the founder of Nexomics Biosciences Inc. The other authors declare that they have no conflicts of interest with the contents of this article.
