# Supplementary material for: ZapG (YhcB/DUF1043), a novel cell division protein in gamma-proteobacteria linking the Z-ring to septal peptidoglycan synthesis
Source: J Biol Chem. 2021 Apr 23;296:100700. doi: 10.1016/j.jbc.2021.100700 (PMC8163987; doi:10.1016/j.jbc.2021.100700)
Supplement: Supporting information [file mmc1.pdf]

YhcB is conserved in proteobacteria

YhcB is conserved across most gamma-proteobacteria but hardly present in any other bacterial clades (**Fig. 1**). Even within gamma-proteobacteria it appears to have been lost secondarily in many clades. While not highly conserved, its amino acid sequence in *E. coli* is 45% and 80% identical to its homologs in *Vibrio* and *Yersinia*, respectively.

In *Escherichia coli*, the *yhcB* gene is located upstream of two periplasmic outer membrane stress sensor (serine) proteases (*degQ* and *degS*) and predicted to be a member of the same transcriptional unit (**Fig. S1**). Upstream of *yhcB*, cell division gene *zapE* (*yhcM*) is encoded on the opposite strand, an arrangement that is conserved across many proteobacteria. Genomically, *yhcB* is clustered with genes involved in outer membrane stress-response and cell division pathways.

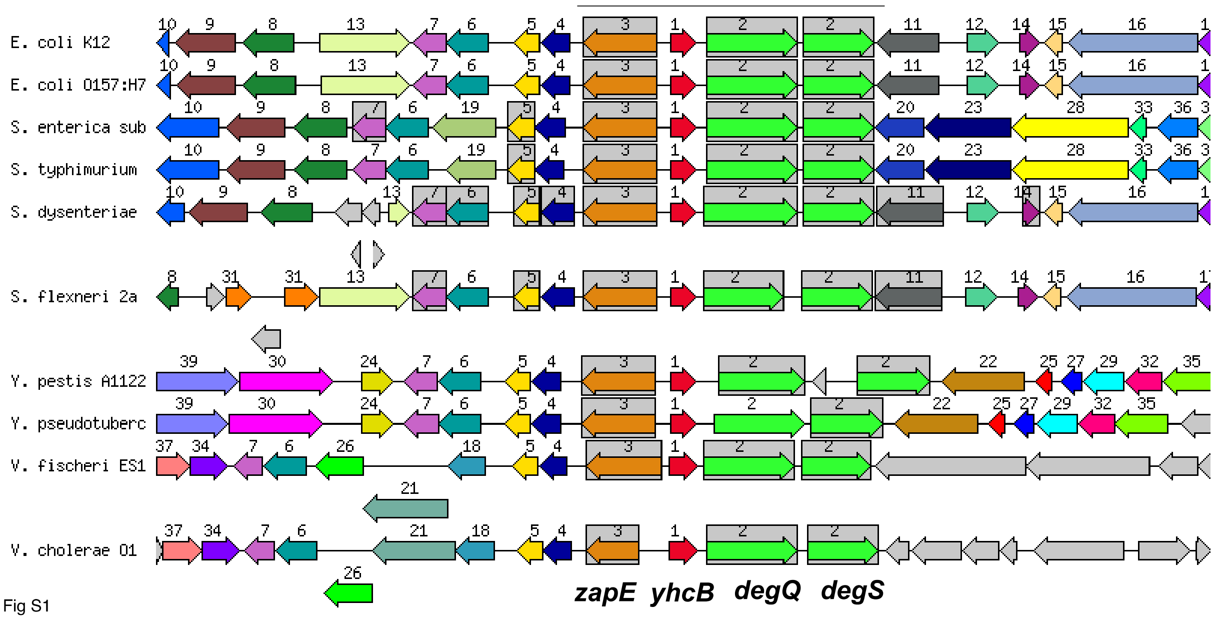

Fig. S1. Gene synteny of *yhcB* and neighboring genes in selected proteobacterial genomes. The homologous genes/ORFs are presented with matching colors and numbers starting with *yhcB* (in red color and # 1). The numbers shown above each ORF are produced by the SEED viewer program. They indicate gene families (homologous genes), but are apparently created individually for each output file, so that they do not represent particular gene family IDs (such as COG IDs). See Overbeek et al. 2013 for details (1).

Phenotypes associated with *yhcB* gene deletion

In order to understand the function and phenotypes of *yhcB*, we utilized a Δ*yhcB* deletion strain to carry out extensive phenotypic testing. In LB at 37°C, Δ*yhcB* grows with a mass doubling time of 25 min, whereas the WT grows with 22 min. Close to stationary phase the Δ*yhcB* cells starts to die. With an optical density difference of 0.3 OD units the difference in survival is more than 17-fold. In LB with 0.5 g salt per liter at 37°C and in minimal glucose medium at 28°C the Δ*yhcB* strain is slightly longer and thinner than the wild type parental strain (**Fig. S2**).

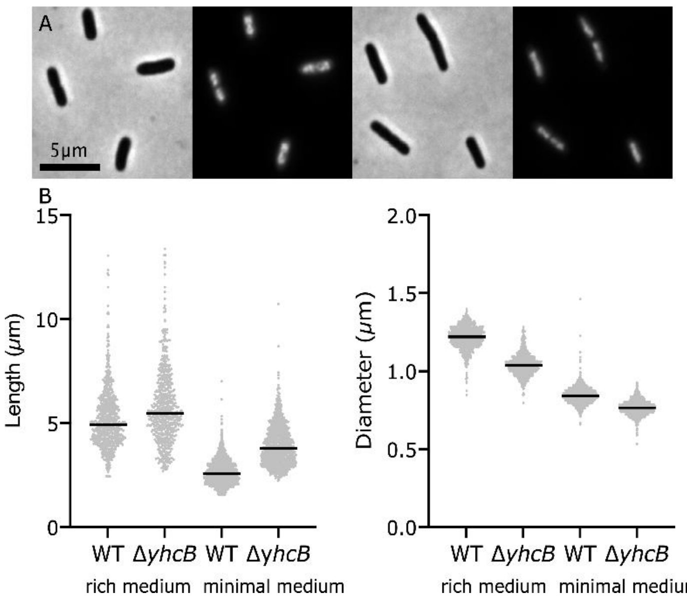

Fig. S2. Imaging of Δ*yhcB* cells. Δ*yhcB* is longer and thinner than its parental strain BW25113 (WT), but nucleoid topography seems to be normal. (**A**). Phase contrast images of the cells and DAPI fluorescence images of the nucleoids of WT cells and Δ*yhcB* cells grown in minimal glucose medium (GB4) at 28°C. The scale bar equals 5 µm. (**B**). Length and diameter of both strains grown in rich medium (LB with 5 g NaCl/L) at 37°C and Gb4 28°C. (C) Demographs of DAPI stained nucleoid distribution in WT (n = 750) (first panel) and Δ*yhcB* (n = 650) cells grown in TY at 37°C (second panel), WT (n = 1521, third panel) and Δ*yhcB* (n = 1095, fourth panel) cells grown Gb4 at 28°C, respectively. The cells are sorted according to cell length and the white outline based on the phase contrast images represents the length of the cells.

The Δ*yhcB* strain grows slowly compared to WT at 37°C and never reaches a max OD_{562} comparable to the parent strain **(Fig S3-a &b)**. Further, the cell envelope integrity in Δ*yhcB* cells was tested using a β-galactosidase assay as described previously (2). Briefly, the cells were transformed with a pCB112 plasmid by mating with an overnight culture of JA200/pCB112 donor strain. The Lac+ exconjugants were selected on LB-Kan-Cam plates and were tested on LB agar plates containing 20 µg/ml CPRG. A light pink color was observed in Δ*yhcB* cells indicating a break down in barrier function of the cell envelope (**Fig S3-c**). The Δ*yhcB* strain also exhibited significantly reduced biofilm formation (**Fig S3-d**). The biofilm formation was determined as described previously (https://www.jove.com/t/2437/microtiter-dish-biofilm-formation-assay). Briefly, the cells were grown in a 96-well plate and the biofilm formation was measured by staining with 0.1 % solution of crystal violet for 15 min at room temperature, followed by washing and quantification of biofilm. The biofilm was quantified by measuring the absorbance at 550 nm and normalized with respect to WT strain.

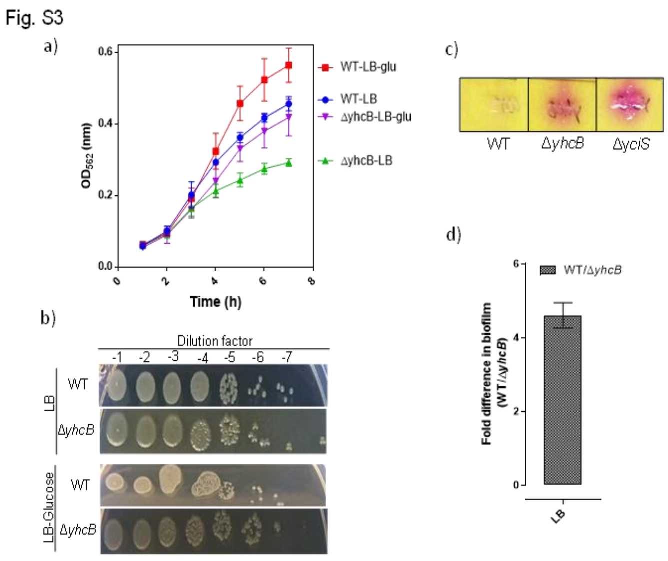

Fig. S3. Phenotypes associated with *yhcB* deletion.
(a) Growth curve/profile of Δ*yhcB* strain in LB and on LB agar. *yhcB* is required for optimal growth of *E. coli*. The Δ*yhcB* strain never reached an OD_{562} comparable to WT strain both in LB and LB-glucose. Data represents at least three independent experiments. (**b**) Serial dilution of Δ*yhcB* in LB and LB-glucose on hard agar plates shows similar growth patterns after 24 hours. (**c**) β-galactosidase (CPRG) assay. Cell envelope integrity of Δy*hcB* strain was tested using a β-galactosidase assay. Both deletion of *yhcB* and an interactor Δ*yciS* showed pinkish colored cells showing defective or permeable cell envelope. **(d)** The Δ*yhcB* cells were found deficient in biofilm formation in LB media. The relative or fold difference in biofilm formation in Δ*yhcB* cells vs WT cells is shown here.

Furthermore, Δ*yhcB* strain showed sensitivity to several cell-division/envelope biogenesis targeting/related antibiotics. Given that we found cell shape and cell division-related phenotypes (e.g. filamented) and susceptibility of Δ*yhcB* strain to several peptidoglycan (PG)-targeting antibiotics (**Fig S4-a**), we tested the effect of cell-wall targeting antibiotics (A22, and Mecillinam) on Δ*yhcB* cells. Proteins of the cell elongasome such as MreB and PBP2 are direct targets of the cell-wall antibiotics A22 and Mecillinam, respectively. A22 (S-[3,4-Dichlorobenzyl] isothiourea) destabilizes MreB filaments (3) and Mecillinam is a β-lactam that specifically inhibits PBP2 (4). We confirmed that the Δ*yhcB* strain was hypersensitive to both A22 and Mecillinam (**Fig 3D**). Furthermore, the Δ*yhcB* cells didn't revive in presence of A22 and Mecillinam whereas Δ*yhcB* cells in absence of antibiotics behaved normally.

Fig S4-a

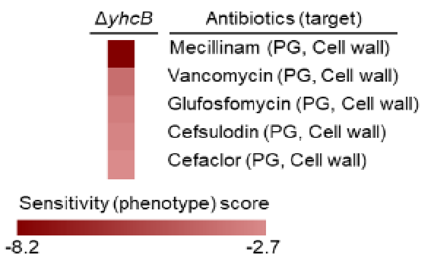

Fig S4-b

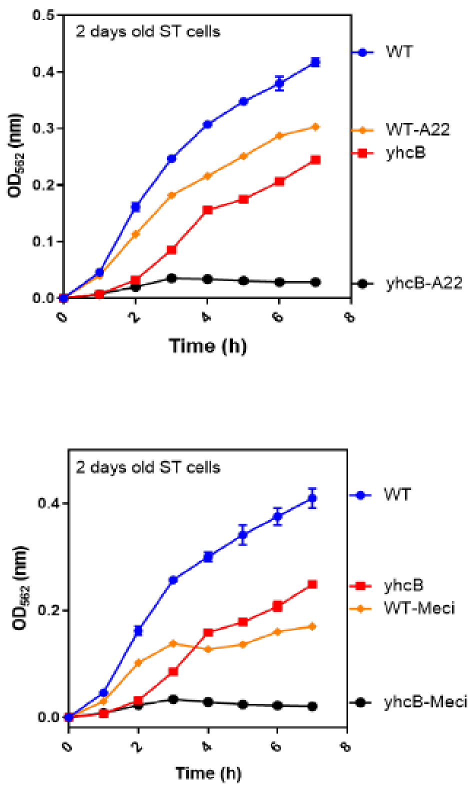

Fig. S4. Hypersensitivity of Δ*yhcB* strain
(a). The susceptibility of an Δ*yhcB* strain to antibiotics targeting the cell wall biogenesis reported in a phenomic profiling of *E. coli* screen.
(b). Hypersensitivity of 2 days old stationary phase Δ*yhcB* cells against cell-wall acting antibiotics. The Δ*yhcB* cells were found hypersensitive to A22 and Mecillinam. The 2-day old stationary phase cells were not able to grow in presence of A22 and Mecillinam. Top=A22, Bottom=Mecillinam; Growth was measured as OD at 562 nm at the given time intervals.

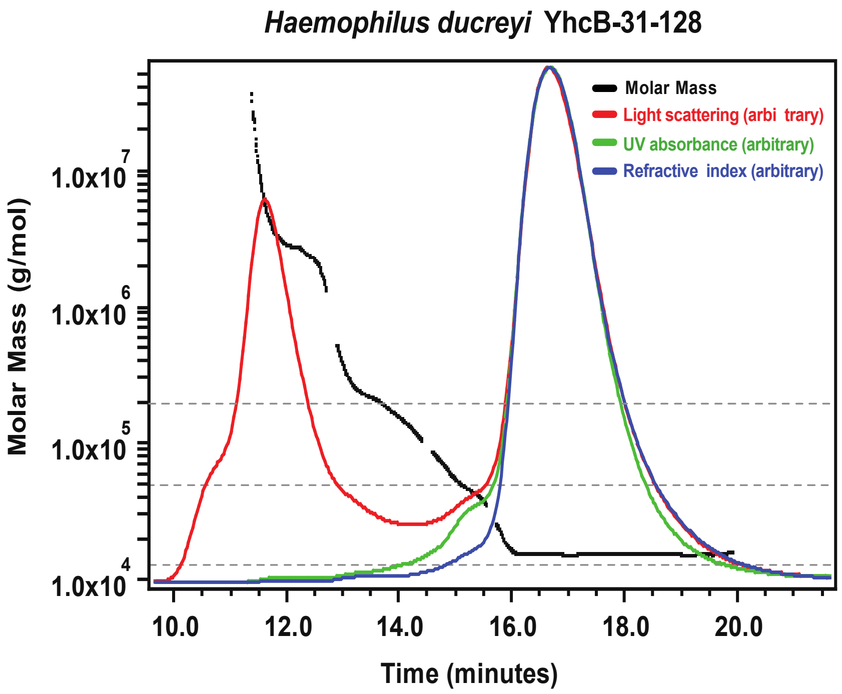

Fig. S5. Size exclusion chromatography and multi-angle light-scattering (SEC-MALS) analysis of the cytosolic segment from *H. ducreyi* YhcB. The analysis was performed on a Shodex KW802.5 column equilibrated in 100 mM NaCl, 5 mM DTT, 20 mM Tris•Cl, pH 7.5. The dotted horizontal lines indicate the predicted molecular weights for a monomer (12,550 daltons), tetramer, and hexadecamer. Quantitative analyses including integration of the refractive index trace (blue) indicate a total recovery of 386 µg of protein distributed between species with average molecular weights of 15.5 kDa (96.3%), 50.2 kDa (2.5%), 199 kDa (0.9%), and 3,520 kDa (0.2%). The molecular weight of the smallest species is 24% higher than the predicted molecular weight for a monomer of this protein construct (12.5 kDa), which could reflect a reversible tendency to oligomerize or, alternative, inaccuracy in the light-scattering-based molecular weight determination in this size range. Reversible oligomerization is concentration-dependent, which generally produces a characteristic parabolic trend in the estimated molecular weight across a SEC peak, with larger values at the center of the peak where the protein concentration is higher compared to is tails. Therefore, the consistency of the calculated molecular weight across the major elution peak suggests the discrepancy in measured vs. predicted molecular weight is more likely to be attributable to inaccurate calibration in this molecular weight range rather than reversible oligomerization. This analysis was performed on the selenomethionine-labeled wild-type protein construct comprising residues 31-128 with an N-terminal methionine and C-terminal affinity tag with sequence LEHHHHHH but without the L51M or L72M mutations used for selenomethionine phasing of the crystal structure.

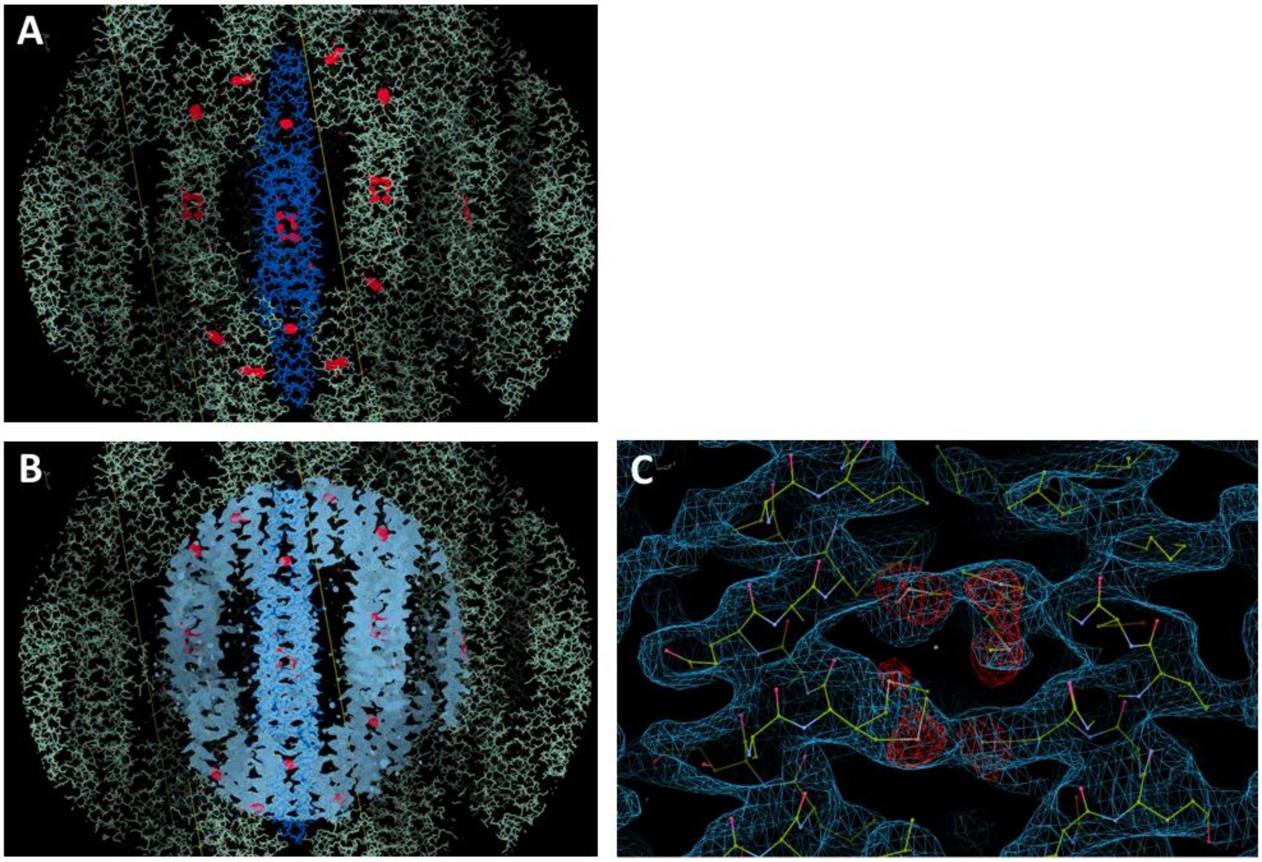

Fig. S6. Electron density maps and lattice packing in the x-ray crystal structure of *Haemophilus ducreyi* YhcB. **(A)** Anomalous difference Fourier map calculated using phases from the final refined model of selenomethionine-labeled *H. ducreyi* I51M-L72M-YhcB. The map contoured at 5 σ is shown in red, the refined atomic model of the tetramer in the asymmetric unit of the crystal structure is shown in blue line representation, its symmetry mates in the crystal lattice are shown in pale green line representation, and the boundaries of the unit cell are shown as yellow lines. The strong peaks in the anomalous difference Fourier map all correspond to selenium atoms in the side chains of the engineered residues Met-51 and Met-72. The latter residue adopts multiple conformations in some subunits in the physiological tetramer. **(B)** The same image in panel **A** but with the addition of the $2f_0$-f_C electron density map calculated from the final refined coordinate model shown in light blue contoured at 1.5 σ. **(C)** Zoomed-in view in a similar orientation of the center of the 4-helix antiparallel coiled-coil bundle where its three orthogonal 2-fold symmetry axes intersect. This view shows that the sidechain of Met-72 adopts two alternative conformations in two of the four subunits, which produces six selenium peaks in this region of the anomalous difference Fourier map rather than four. The anomalous map (red) is contoured at 4.2 σ, while the $2f_0$-f_C map (blue) is contoured at 0.26 σ in this view. The refined crystallographic coordinate model is shown in ball-and-stick representation with carbon in yellow, oxygen in red, nitrogen in blue, and selenium in gray.

Evolutionary coupling probabilities for Fig. 8D (main text).

i	j	i_id	j_id	r_sco	s_sco	prob
70	76	70_Y	76_H	0.1611	2.721	1.000
56	81	56_F	81_S	0.1315	2.222	0.998
34	44	34_L	44_E	0.1174	1.983	0.993
73	119	73_L	119_E	0.1037	1.752	0.979
59	115	59_S	115_R	0.1033	1.746	0.978
10	16	10_L	16_I	0.0998	1.687	0.971
15	109	15_I	109_A	0.0984	1.663	0.968
35	39	35_Q	39_E	0.0907	1.533	0.942
48	52	48_Y	52_L	0.0852	1.439	0.913
62	70	62_L	70_Y	0.0851	1.438	0.913
60	76	60_A	76_H	0.0849	1.435	0.912
13	115	13_G	115_R	0.0843	1.425	0.908
59	70	59_S	70_Y	0.0830	1.403	0.899
120	123	120_G	123_G	0.0827	1.397	0.897
37	57	37_E	57_A	0.0808	1.365	0.882
47	50	47_E	50_E	0.0783	1.324	0.860
74	81	74_Y	81_S	0.0773	1.306	0.850
13	114	13_G	114_P	0.0770	1.301	0.847
14	17	14_I	17_G	0.0754	1.275	0.831
13	116	13_G	116_D	0.0745	1.259	0.820
112	129	112_Q	129_A	0.0741	1.253	0.816
8	54	8_I	54_S	0.0740	1.251	0.814
33	49	33_A	49_R	0.0736	1.243	0.808
118	124	118_S	124_L	0.0735	1.242	0.808
9	63	9_G	63_L	0.0734	1.241	0.807
52	73	52_L	73_L	0.0734	1.240	0.806
49	113	49_R	113_M	0.0732	1.236	0.803
49	82	49_R	82_S	0.0713	1.205	0.779
42	74	42_K	74_Y	0.0706	1.194	0.770
67	73	67_A	73_L	0.0697	1.178	0.757
19	57	19_V	57_A	0.0697	1.177	0.756
49	112	49_R	112_Q	0.0693	1.171	0.751
19	96	19_V	96_R	0.0683	1.154	0.736
48	70	48_Y	70_Y	0.0682	1.153	0.735
6	9	6_A	9_G	0.0679	1.148	0.730
67	74	67_A	74_Y	0.0672	1.136	0.719
124	127	124_L	127_T	0.0671	1.134	0.717
62	84	62_L	84_L	0.0669	1.130	0.713
65	69	65_T	69_D	0.0668	1.128	0.711
60	78	60_A	78_A	0.0667	1.128	0.711
79	82	79_K	82_S	0.0666	1.125	0.708
114	117	114_P	117_Y	0.0664	1.122	0.706

48	62	48_Y	62_L	0.0660	1.115	0.699
66	72	66_M	72_Q	0.0659	1.113	0.697
8	78	8_I	78_A	0.0658	1.112	0.696
56	60	56_F	60_A	0.0656	1.108	0.692
65	99	65_T	99_L	0.0654	1.104	0.688
103	107	103_E	107_D	0.0654	1.104	0.688
8	65	8_I	65_T	0.0650	1.098	0.682
6	10	6_A	10_L	0.0648	1.095	0.679
56	78	56_F	78_A	0.0645	1.090	0.673
5	9	5_Y	9_G	0.0641	1.083	0.666
59	63	59_S	63_L	0.0638	1.078	0.661
55	76	55_H	76_H	0.0637	1.076	0.659
27	39	27_K	39_E	0.0632	1.068	0.650
40	46	40_K	46_D	0.0629	1.063	0.645
3	17	3_W	17_G	0.0628	1.061	0.643
41	118	41_N	118_S	0.0625	1.056	0.638
61	71	61_E	71_R	0.0623	1.053	0.634
11	109	11_V	109_A	0.0623	1.052	0.633
34	41	34_L	41_N	0.0622	1.050	0.631
4	25	4_E	25_N	0.0613	1.036	0.616
47	51	47_E	51_E	0.0610	1.031	0.610
125	128	125_L	128_G	0.0610	1.031	0.610
6	23	6_A	23_F	0.0610	1.031	0.610
22	38	22_R	38_L	0.0610	1.031	0.610
106	112	106_N	112_Q	0.0608	1.027	0.606
63	77	63_L	77_M	0.0607	1.026	0.605
55	84	55_H	84_L	0.0606	1.024	0.602
64	77	64_D	77_M	0.0602	1.017	0.594
41	48	41_N	48_Y	0.0597	1.009	0.585
25	29	25_N	29_R	0.0596	1.007	0.583
4	54	4_E	54_S	0.0593	1.003	0.579
37	42	37_E	42_K	0.0590	0.997	0.572
15	19	15_I	19_V	0.0586	0.990	0.564
9	60	9_G	60_A	0.0580	0.980	0.552
18	21	18_A	21_M	0.0578	0.977	0.549
21	125	21_M	125_L	0.0578	0.977	0.549
120	125	120_G	125_L	0.0577	0.975	0.546
10	17	10_L	17_G	0.0575	0.971	0.542
81	118	81_S	118_S	0.0565	0.955	0.523
13	56	13_G	56_F	0.0565	0.955	0.523
114	118	114_P	118_S	0.0564	0.953	0.521
75	93	75_Q	93_N	0.0560	0.946	0.513
48	76	48_Y	76_H	0.0557	0.941	0.507
107	111	107_D	111_V	0.0556	0.939	0.504

63	75	63_L	75_Q	0.0555	0.938	0.503
11	57	11_V	57_A	0.0550	0.930	0.494
13	32	13_G	32_Q	0.0549	0.927	0.490
8	12	8_I	12_V	0.0549	0.927	0.490
35	68	35_Q	68_H	0.0547	0.925	0.488
47	120	47_E	120_G	0.0544	0.919	0.481
102	105	102_S	105_S	0.0544	0.919	0.481
22	31	22_R	31_Q	0.0543	0.917	0.479
90	108	90_A	108_Q	0.0542	0.916	0.477
41	45	41_N	45_L	0.0540	0.913	0.474
59	67	59_S	67_A	0.0540	0.912	0.473
14	115	14_I	115_R	0.0539	0.911	0.472
85	113	85_L	113_M	0.0537	0.907	0.467
7	28	7_L	28_L	0.0536	0.906	0.466
70	74	70_Y	74_Y	0.0532	0.899	0.458
33	83	33_A	83_S	0.0531	0.897	0.455
68	92	68_H	92_A	0.0531	0.897	0.455
3	86	3_W	86_P	0.0530	0.896	0.454
22	115	22_R	115_R	0.0530	0.896	0.454
50	77	50_E	77_M	0.0530	0.895	0.453
18	24	18_A	24_G	0.0529	0.893	0.451
115	119	115_R	119_E	0.0527	0.891	0.448
114	123	114_P	123_G	0.0526	0.889	0.446
35	94	35_Q	94_P	0.0525	0.888	0.445
69	86	69_D	86_P	0.0524	0.886	0.443
7	113	7_L	113_M	0.0523	0.883	0.439
95	123	95_F	123_G	0.0523	0.883	0.439
110	129	110_P	129_A	0.0522	0.881	0.437
45	73	45_L	73_L	0.0521	0.880	0.436
6	109	6_A	109_A	0.0520	0.879	0.435
55	70	55_H	70_Y	0.0520	0.878	0.433
7	14	7_L	14_I	0.0519	0.877	0.432
38	70	38_L	70_Y	0.0513	0.866	0.420
57	74	57_A	74_Y	0.0511	0.863	0.416
11	15	11_V	15_I	0.0510	0.861	0.414
16	41	16_I	41_N	0.0508	0.858	0.411
31	55	31_Q	55_H	0.0508	0.858	0.411
40	127	40_K	127_T	0.0508	0.858	0.411
49	111	49_R	111_V	0.0508	0.858	0.411
16	65	16_I	65_T	0.0506	0.855	0.407
123	126	123_G	126_R	0.0505	0.853	0.405
13	81	13_G	81_S	0.0504	0.851	0.403
69	74	69_D	74_Y	0.0504	0.851	0.403
59	113	59_S	113_M	0.0503	0.850	0.402

3	87	3_W	87_E	0.0502	0.848	0.399
2	48	2_T	48_Y	0.0502	0.847	0.398
116	123	116_D	123_G	0.0501	0.847	0.398
112	120	112_Q	120_G	0.0500	0.845	0.396
47	54	47_E	54_S	0.0499	0.843	0.394
46	50	46_D	50_E	0.0499	0.843	0.394
75	96	75_Q	96_R	0.0498	0.842	0.393
98	101	98_R	101_E	0.0498	0.841	0.392
48	55	48_Y	55_H	0.0494	0.834	0.384
57	71	57_A	71_R	0.0493	0.834	0.384
32	42	32_Q	42_K	0.0493	0.833	0.383
52	69	52_L	69_D	0.0492	0.832	0.382
80	125	80_S	125_L	0.0491	0.829	0.378
48	53	48_Y	53_V	0.0491	0.829	0.378
64	68	64_D	68_H	0.0491	0.829	0.378
58	98	58_R	98_R	0.0490	0.828	0.377
6	44	6_A	44_E	0.0489	0.827	0.376
66	74	66_M	74_Y	0.0488	0.825	0.374
7	75	7_L	75_Q	0.0487	0.823	0.372
26	82	26_R	82_S	0.0487	0.823	0.372
64	74	64_D	74_Y	0.0486	0.822	0.371
97	101	97_N	101_E	0.0486	0.821	0.369
87	119	87_E	119_E	0.0486	0.821	0.369
117	123	117_Y	123_G	0.0486	0.820	0.368
26	32	26_R	32_Q	0.0486	0.820	0.368
44	96	44_E	96_R	0.0486	0.820	0.368
12	54	12_V	54_S	0.0485	0.819	0.367
34	113	34_L	113_M	0.0485	0.819	0.367
33	118	33_A	118_S	0.0485	0.819	0.367
40	94	40_K	94_P	0.0484	0.818	0.366
33	51	33_A	51_E	0.0484	0.818	0.366
9	15	9_G	15_I	0.0483	0.816	0.364
48	113	48_Y	113_M	0.0483	0.816	0.364
8	97	8_I	97_N	0.0481	0.814	0.362
11	104	11_V	104_A	0.0480	0.811	0.359
26	64	26_R	64_D	0.0479	0.809	0.356
12	15	12_V	15_I	0.0478	0.807	0.354
77	89	77_M	89_S	0.0478	0.807	0.354
32	86	32_Q	86_P	0.0478	0.807	0.354
16	102	16_I	102_S	0.0477	0.807	0.354
100	105	100_A	105_S	0.0477	0.806	0.353
10	99	10_L	99_L	0.0477	0.805	0.352
24	72	24_G	72_Q	0.0476	0.805	0.352
40	44	40_K	44_E	0.0476	0.805	0.352

56	117	56_F	117_Y	0.0476	0.805	0.352
44	97	44_E	97_N	0.0474	0.802	0.349
81	95	81_S	95_F	0.0474	0.800	0.347
42	79	42_K	79_K	0.0472	0.798	0.345
59	76	59_S	76_H	0.0472	0.797	0.344
12	61	12_V	61_E	0.0471	0.797	0.344
23	73	23_F	73_L	0.0470	0.795	0.342
80	119	80_S	119_E	0.0470	0.794	0.341
127	130	127_T	130_K	0.0469	0.793	0.340
52	93	52_L	93_N	0.0469	0.792	0.338
52	86	52_L	86_P	0.0467	0.789	0.335
107	120	107_D	120_G	0.0467	0.788	0.334
3	72	3_W	72_Q	0.0465	0.785	0.331
51	69	51_E	69_D	0.0462	0.781	0.327
37	55	37_E	55_H	0.0462	0.781	0.327
27	34	27_K	34_L	0.0461	0.779	0.325
90	103	90_A	103_E	0.0461	0.778	0.324
21	123	21_M	123_G	0.0460	0.777	0.323
5	22	5_Y	22_R	0.0459	0.775	0.321
10	24	10_L	24_G	0.0459	0.775	0.321
18	28	18_A	28_L	0.0457	0.773	0.319

Supplementary references.

1. Overbeek, R., Olson, R., Pusch, G. D., Olsen, G. J., Davis, J. J., Disz, T., Edwards, R. A., Gerdes, S., Parrello, B., Shukla, M., Vonstein, V., Wattam, A. R., Xia, F., and Stevens, R. (2014) The SEED and the Rapid Annotation of microbial genomes using Subsystems Technology (RAST). *Nucleic Acids Res.* 10.1093/nar/gkt1226

2. Paradis-Bleau, C., Kritikos, G., Orlova, K., Typas, A., and Bernhardt, T. G. (2014) A Genome-Wide Screen for Bacterial Envelope Biogenesis Mutants Identifies a Novel Factor Involved in Cell Wall Precursor Metabolism. *PLoS Genet.* **10**, e1004056

3. van den Ent, F., Izoré, T., Bharat, T. A. M., Johnson, C. M., and Löwe, J. (2014) Bacterial actin MreB forms antiparallel double filaments. *Elife*. **3**, e02634

4. Cho, H., Uehara, T., and Bernhardt, T. G. (2014) Beta-Lactam Antibiotics Induce a Lethal Malfunctioning of the Bacterial Cell Wall Synthesis Machinery. *Cell*. **159**, 1300–1311
